# An analytical theory of curriculum learning in teacher–student networks* *This article was also accepted in NeurIPS2022 Proceedings.


**DOI:** 10.1088/1742-5468/ac9b3c

**Published:** 2022-11-24

**Authors:** Luca Saglietti, Stefano Sarao Mannelli, Andrew Saxe

**Affiliations:** 1 Institute for Data Science and Analytics, Bocconi University, Italy; 2 Gatsby Computational Neuroscience Unit and Sainsbury Wellcome Centre, University College, London, United Kingdom; 3 FAIR, Meta AI, United States of America

**Keywords:** machine learning

## Abstract

In animals and humans, curriculum learning—presenting data in a curated order—is critical to rapid learning and effective pedagogy. A long history of experiments has demonstrated the impact of curricula in a variety of animals but, despite its ubiquitous presence, a theoretical understanding of the phenomenon is still lacking. Surprisingly, in contrast to animal learning, curricula strategies are not widely used in machine learning and recent simulation studies reach the conclusion that curricula are moderately effective or even ineffective in most cases. This stark difference in the importance of curriculum raises a fundamental theoretical question: when and why does curriculum learning help? In this work, we analyse a prototypical neural network model of curriculum learning in the high-dimensional limit, employing statistical physics methods. We study a task in which a sparse set of informative features are embedded amidst a large set of noisy features. We analytically derive average learning trajectories for simple neural networks on this task, which establish a clear speed benefit for curriculum learning in the online setting. However, when training experiences can be stored and replayed (for instance, during sleep), the advantage of curriculum in standard neural networks disappears, in line with observations from the deep learning literature. Inspired by synaptic consolidation techniques developed to combat catastrophic forgetting, we propose curriculum-aware algorithms that consolidate synapses at curriculum change points and investigate whether this can boost the benefits of curricula. We derive generalisation performance as a function of consolidation strength (implemented as an *L*_2_ regularisation/elastic coupling connecting learning phases), and show that curriculum-aware algorithms can yield a large improvement in test performance. Our reduced analytical descriptions help reconcile apparently conflicting empirical results, trace regimes where curriculum learning yields the largest gains, and provide experimentally-accessible predictions for the impact of task parameters on curriculum benefits. More broadly, our results suggest that fully exploiting a curriculum may require explicit adjustments in the loss.

## 1. Introduction

Presenting learning materials in a meaningful order according to a curriculum greatly helps learning in animals and humans [1–4], and is considered an essential aspect of good pedagogy [5]. For example, humans have been shown to learn visual discriminations faster when presented with examples that exaggerate the relevant difference between classes, a phenomenon known as ‘fading’ [6–8]. Beyond humans, curricula in the form of ‘shaping’ or ‘staircase’ procedures are a near-universal feature of task designs in animal studies, without which training often fails entirely. For instance, the International Brain Laboratory task, a standardised perceptual decision-making training paradigm in mice, involves six stages of increasing difficulty before reaching final performance [9].

Building from this intuition, a seminal series of papers proposed a similar curriculum learning approach for machine learning (ML) [10–12]. In striking contrast to the clear benefits of curriculum in biological systems, however, curriculum learning has generally yielded equivocal benefits in artificial systems. Experiments in a variety of domains [13, 14] have found usually modest speed and generalisation improvements from curricula. Recent extensive empirical analyses have found minimal benefits on standard datasets [15]. Indeed, a common intuition in deep learning practice holds that training distributions should ideally be as close as possible to testing distributions, a notion which runs counter to curriculum. Perhaps the only areas where curricula are actively used are in large language models [16] and certain reinforcement learning settings [17].

This gap between the effect of curriculum in biological and artificial learning systems poses a puzzle for theory. When and why is curriculum learning useful? What properties of a task determine the extent of possible benefits? What ordering of learning material is most beneficial? And can new learning algorithms better exploit curricula? Compared to the empirical investigations of curriculum learning, theoretical results on curriculum learning remain sparse. Most notably, [18, 19] show that curriculum can lead to faster learning in a simple setting, but the effects of curriculum on asymptotic generalisation and the dependence on task structure remain unclear. A hint that indeed curriculum learning might lead to statistically different minima comes from a connection between constraint-satisfaction problems and physics results on flow networks [20], but to our knowledge no direct result has been reported in the modern theoretical ML literature.

In this work we study the impact of curriculum using the analytically tractable teacher–student framework and the tools of statistical physics [21–24]. High-dimensional teacher–student models are a popular approach for systematically studying learning behaviour in neural networks [22, 25, 26], and have recently been leveraged to analyse a variety of phenomena [27–32]. Using a simple model to build structured data [12], we examine the impact of ordering examples by increasing difficulty (curriculum), decreasing difficulty (anti-curriculum), or standard shuffled training. We derive exact expressions for the online learning dynamics and the performance of batch learning. However, in the latter, curriculum confers no benefit under standard training. Motivated by theories of synaptic consolidation and elastic weight consolidation [33, 34], we introduce elastic penalties (Gaussian priors) that regularise training toward solutions obtained in prior curriculum phases. With these priors, curriculum yields benefits both in the online 3 and in the batch 4 settings.

## 2. Model definition and overview of approach

**Figure 1.**
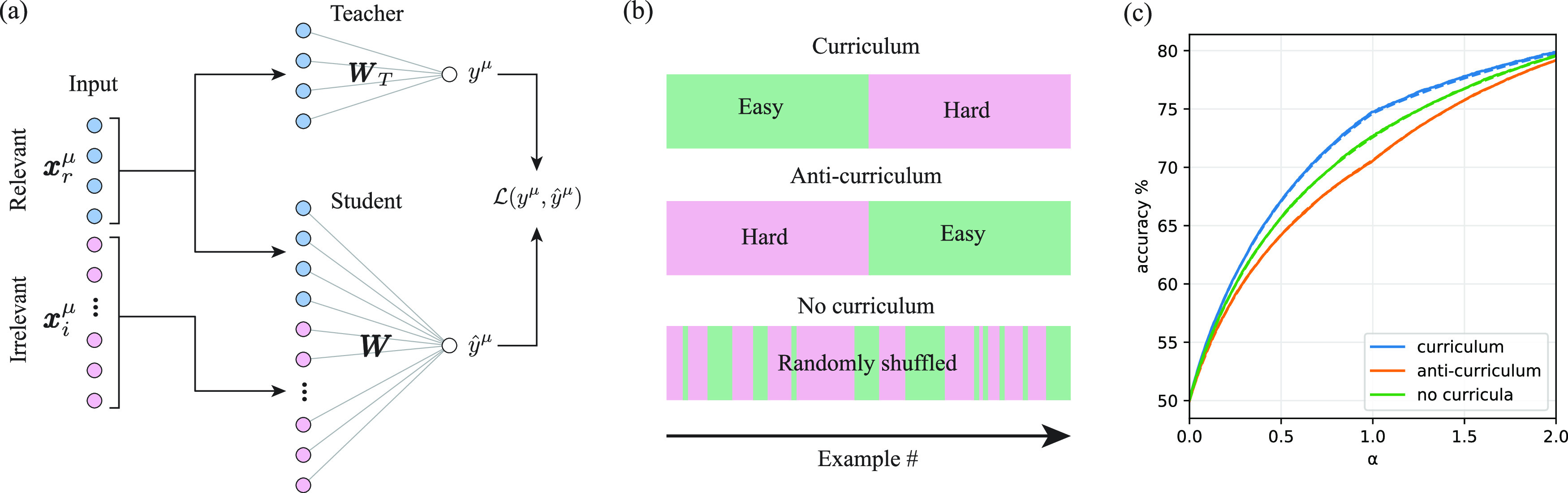
Teacher–student setting for curriculum learning. (a) Illustration of teacher–student setting in which a ‘student’ network is trained from i.i.d. inputs with labels from a ‘teacher’ network. Since the teacher network is sparse, its output depends only on a subset of *relevant* input features. (b) We consider curricula which order examples by difficulty, here taken to be the variance in the irrelevant feature dimensions. We refer to increasing, decreasing, and random difficulty order as curriculum, anti-curriculum, and no curriculum, respectively. (c) Example test error on hard examples for the student over training. The switch-point between easy and hard samples lies at *α* = 1/2. Solid lines show numerical simulations, while dashed lines show theoretical predictions derived in section 3. For this particular parameter setting, curriculum speeds learning but only modestly improves final performance at *α* = 1. Parameters: *α*_1_ = 1, *α*_2_ = 1, Δ_1_ = 0, Δ_2_ = 1, *γ* = 10^−5^, *η* = 3.

In the following, we revisit a prototypical model of curriculum learning from [12] that finds correspondence to the fading literature [6] as highlighted in section 5. Our setting is summarised in figure 1. The model entails a simple teacher–student setup, where teacher and student are each shallow one-layer neural networks of size *N* (also known as perceptrons). The learning task for the student is a binary classification problem, with dataset $\mathcal{D}={\left\{({y}^{\mu },{\boldsymbol{x}}^{\,\mu })\right\}}_{\mu =1}^{M}$, where the ground-truth labels are produced by the teacher network *y* ^*μ*^ = sign ***W***_*T*_ ⋅ ***x*** ^*μ*^. A key feature of this model is that the teacher network is sparse, with only a fraction *ρ* < 1 of $\sim \mathcal{N}(0,1)$ non-zero components. Therefore, in order to achieve a good test accuracy, the student has to guess which components should be set to zero and align the relevant weights in the correct direction. A large range of 0 < *ρ* < 1 could give rise to the phenomenology we seek to analyse. In the remainder of the paper we will focus on the case *ρ* = 0.5.

We model the variable degree of difficulty in the samples by decomposing each input vector as ${\boldsymbol{x}}^{\,\mu }=[{\boldsymbol{x}}_{r}^{\,\mu },{\boldsymbol{x}}_{i}^{\,\mu }]\in {\mathbb{R}}^{N}$, where ${\boldsymbol{x}}_{r}^{\,\mu }\in {\mathbb{R}}^{\rho N}$ denotes the relevant components of the input, and ${\boldsymbol{x}}_{i}^{\mu }\in {\mathbb{R}}^{(1-\rho )N}$ the irrelevant ones. Note that, crucially, the sparse teacher network is completely blind to the irrelevant part of the input: ${y}^{\mu }=\mathrm{sign}\ {\sum }_{j=1}^{\rho N}\,{W}_{T,j}{x}_{r,j}^{\mu }$. While ${x}_{r,j}^{\mu }$ i.i.d. $\mathcal{N}(0,1),\forall \enspace \mu $,^5^ 5In [12] the distribution of relevant and irrelevant inputs is uniform between 0 and 1, but this difference does not qualitatively change the results. we consider the variance for the irrelevant components to be sample-dependent ${x}_{i,j}^{\mu }\sim \mathcal{N}(0,{{\Delta}}^{\mu })$. Note that a smaller variance in the irrelevant part induces a higher SNR in the student learning problem.

The dataset is partitioned according to difficulty levels given by the variances of the irrelevant inputs. For simplicity we consider only two partitions in most of our analysis, but generalisations to more difficulty levels follow straightforwardly. We have a dataset with *M* = (*α*_1_ + *α*_2_)*N* = *αN* total samples, in which the irrelevant inputs of the first *α*_1_ *N* samples have variance Δ_1_, and the remaining *α*_2_ *N* samples have variance Δ_2_ > Δ_1_. In the curriculum learning condition we present the easy examples first, while in the anti-curriculum condition we present the hard examples first. Standard learning presents examples shuffled in random order.

## 3. Online dynamical solution in the large input limit

We start by focusing on the same online learning setting explored in [12]. We consider a one-layer student network with sigmoidal activation function, $\sigma (\cdot )=\mathrm{e}\mathrm{r}\mathrm{f}(\cdot /\sqrt{2})$, that learns to minimise a mean square error loss with *L*_2_ regularisation of intensity *γ*, using gradient descent. This yields the updates

\begin{equation*}{\boldsymbol{W}}^{\mu +1}\!\!\!=\!\!{\boldsymbol{W}}^{\mu }\!-\!\frac{\eta }{\sqrt{N}}{\sigma }^{\prime }\!\left(\!\frac{{\boldsymbol{W}}^{\mu }\!\cdot \!{\boldsymbol{x}}^{\mu }}{\sqrt{N}}\!\right)\!\left(\!\sigma \!\left(\frac{{\boldsymbol{W}}^{\mu }\!\cdot \!{\boldsymbol{x}}^{\mu }}{\sqrt{N}}\right)\!-\!{y}^{\mu }\!\right){\boldsymbol{x}}^{\mu }\!-\!\gamma {\boldsymbol{W}}^{\mu }.\end{equation*}

The dynamics of the model can be analysed in the high-dimensional limit *N*, *M* → ∞ with $\alpha =M/N=\mathcal{O}(1)$. Generalising the results of [26, 35] on the online stochastic gradient descent dynamics in single-layer regression problems, we obtain a precise description of the performance at all times, as a function of several order parameters: the squared norm of the relevant and irrelevant part of the student weights ${Q}_{r}=\frac{1}{N}{\boldsymbol{W}}^{r}\cdot {\boldsymbol{W}}^{r}$ and ${Q}_{i}=\frac{1}{N}{\boldsymbol{W}}^{i}\cdot {\boldsymbol{W}}^{i}$, respectively; the overlap of the relevant weights of the student and teacher $R=\frac{1}{N}{\boldsymbol{W}}^{r}\cdot {\boldsymbol{W}}_{T}$; and the squared norm of the teacher vector $T=\frac{1}{N}{\boldsymbol{W}}_{T}\cdot {\boldsymbol{W}}_{T}$. In particular, given *Q*_*r*_, *Q*_*i*_, *R* and *T*, the test loss (i.e. average loss on a new example) on a dataset with variance Δ in the irrelevant inputs is given by

\begin{align*}\hfill {\mathcal{L}}_{\text{MSE}}\!&amp; =\!\frac{1}{2}\!+\!\frac{1}{\pi }{\sin }^{-1}\!\!\frac{{Q}_{r}+{\Delta}{Q}_{i}}{1+{Q}_{r}+{\Delta}{Q}_{i}}\!-\!\frac{2}{\pi }{\sin }^{-1}\!\!\frac{R/\sqrt{T}}{\sqrt{{Q}_{r}+{\Delta}{Q}_{i}+1}},\hfill \end{align*}

the accuracy by

\begin{equation*}\quad \mathcal{A}=\mathbb{E}\left[\frac{1}{2}(y\ \mathrm{sign}\hat{y}+1)\right]=\frac{1}{2}+\frac{1}{\pi }{\sin }^{-1}\left(\frac{R}{\sqrt{T({Q}_{r}+{\Delta}{Q}_{i})}}\right).\end{equation*}

If the dataset contains a random mixture of different difficulty levels Δ_1_, Δ_2_, …, the loss and accuracy can be obtained by taking a weighted average over the partitions.

To understand how test performance changes through learning, we study the evolution of the order parameters. Combining their definition with the definition of the dynamics (1) and the fact that the random variables concentrate in the high-dimension as *N* → ∞, we obtain an analytic form for the updates: ${Q}_{r}{\leftarrow}{f}_{{Q}_{r}}\left({Q}_{r},{Q}_{i},R,T\right),{Q}_{i}{\leftarrow}{f}_{{Q}_{i}}\left({Q}_{r},{Q}_{i},R,T\right),R{\leftarrow}{f}_{R}\left({Q}_{r},{Q}_{i},R,T\right);$ where ${f}_{{Q}_{r}}$, ${f}_{{Q}_{i}}$ and *f*_*R*_ are long but explicit expressions that are reported in the appendices.

*Dynamical advantages of curriculum*. With these theoretical results in hand, we now characterise the performance of curricula in the online setting. The dynamical equations have two key advantages relative to simulating models in this setting. First, they are free of finite size effects and stochastic fluctuations. And second, their evaluation is very fast, enabling systematic exploration of the parameter space of the problem, along with fine-grained optimisation over hyper-parameters such as learning rate, weight decay and scaling in the initialisation.

Optimising final test accuracy separately for each curriculum strategy, we find that curriculum learning is the optimal strategy, followed by baseline (no-curriculum) and lastly anti-curriculum. In figure 1(c) we show typical learning trajectories for a dataset with equal numbers of easy and hard samples. The results of the simulations (solid lines) are well-described by our theoretical equations (dashed lines), and show that the curriculum strategy leads to better performance throughout training. Figure 1(c) shows the evolution during training of the test accuracy computed on the whole dataset.

**Figure 2.**
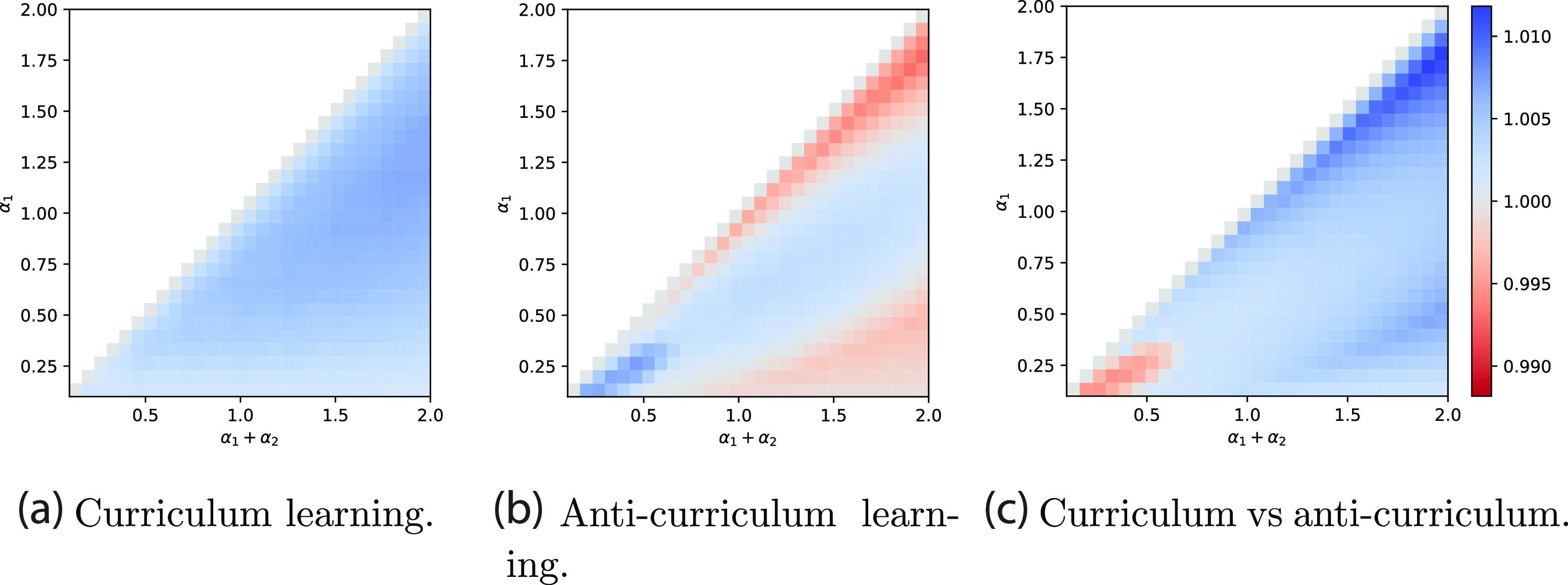
Phase diagram of online learning performance gap with optimal parameters. The colour scale shows the ratio of the accuracy on hard instances reached by curriculum over no-curriculum (a), anti-curriculum over no-curriculum (b), and curriculum over anti-curriculum (c), as a function of the total dataset size (*α*_1_ + *α*_2_) and easy dataset size (*α*_1_). Curriculum broadly benefits performance and anti-curriculum is effective in certain regions, but the size of the improvement is modest. Parameters: *ρ* = 0.50, Δ_1_ = 0, Δ_2_ = 1.

Next we systematically trace the effect of curriculum for a range of total dataset sizes (*α*_1_ + *α*_2_) and number of easy examples *α*_1_ in the phase diagram in figure 2. This diagram shows on the left (centre) the accuracy on hard instances reached at the end of training by curriculum learning (anti-curriculum learning respectively) normalised by the accuracy reached by the standard strategy. The two heatmaps show that curriculum learning always outperforms standard learning and that, on the other hand, anti-curriculum learning outperforms standard learning only in part of the diagram. Comparing the two strategies, figure 2 (right), we can observe that there is a region for small *α* and *α*_1_ where anti-curriculum learning is the best strategy, while in the majority of the situations curriculum learning is the best strategy. Interestingly, there is a sizeable region of the diagram in which *both* curriculum and anti-curriculum help, possibly explaining why both have been recommended in prior work [12, 14, 36–38]. A possible intuition behind this counter-intuitive phonemenon highlighted by our analysis, is that, in some settings, the large amount of noise contained in the hard data will always be too disruptive for effective learning. Thus, leaving the easy (cleaner) data for last could allow the model to better exploit the easy data.

Further, we find that our setting, in which a small task-relevant signal is embedded in large task-irrelevant variation, is critical to the benefit of curriculum. Figure 4 shows performance as a function of sparsity *ρ*, additional details are deferred in appendix C. Non-sparse tasks do not benefit. Hence curriculum aids tasks with many irrelevant factors of variation. Interestingly, the literature from human psychology shows precisely this: no curriculum benefits for low-dimensional tasks or tasks with no variation in irrelevant dimensions [6].

**Figure 3.**
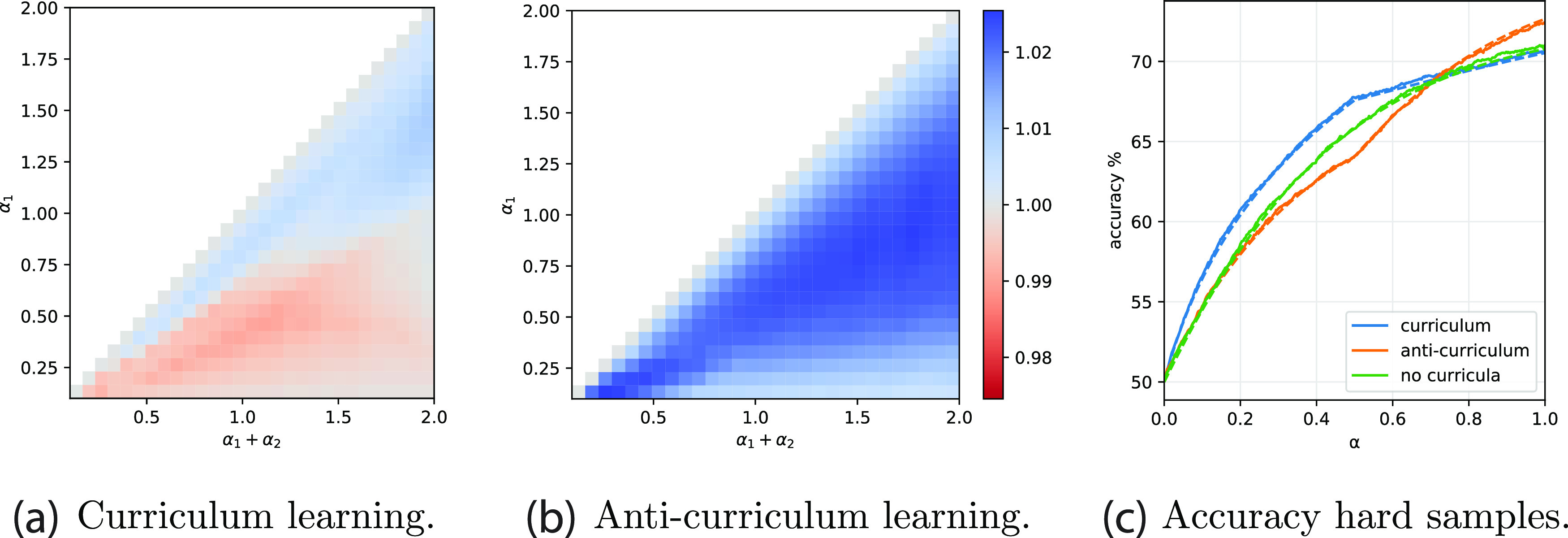
Performance gap starting from high initialisation variance. The first two figures show the accuracy-gap on hard instances between curriculum learning and the baseline (a) and anti-curriculum learning and the baseline (b). Contrary to the phase diagram in figure 2, curriculum learning is not always the optimal and anti-curriculum is not always the worst strategy. The right panel shows the accuracy evaluated on the hard samples for *α*_1_ = *α*_2_ = 0.5.

Our results also highlight the intricate dependence of curriculum on parameters of the learning setup. If not all parameters are correctly optimised, we can observe more complex scenarios. For instance, anti-curriculum learning is always the best strategy starting from a large variance in the weights’ distribution, as figure 3 shows for weights of order 1. In this case, curriculum learning shows an advantage only in the first phase when easy examples are shown, which is consistent with the results of [19]. However, in the next phase when hard examples are shown, the curriculum strategy does not extract enough information and it is outperformed by the other two strategies. The fact that curriculum or anti-curriculum can look better depending on other learning parameters like initialisation might help explain the confusion in the literature over the best protocol [12, 14, 36–38]. At least in this model, better performance from anti-curriculum is a signature of a sub-optimal choice of the parameters. To summarise our findings in this online learning setting, curriculum mainly offers a *dynamical advantage*: it speeds learning, with only minimal impact on asymptotic performance.

## 4. Batch learning solution

The previous section discussed the online case where each example is used once and then discarded. However, in common ML practice, neural networks typically revisit each sample repeatedly until convergence. Therefore an important question is: *can curricula lead to a generalisation improvement when trained on the same dataset until convergence?*

We investigate this question by considering a student that learns from slices of a dataset in distinct optimisation phases, where in each phase the student optimises a *L*_2_-regularised logistic loss. Without further modification, curriculum can have no effect in this setting: due to the convex nature of the teacher–student setup [22], the network is bound to converge to a minimum uniquely determined by the final slice of data, with no memory of the progress made at intermediate steps. This simple observation may help explain empirical observations on real data, such as [15], which find no benefit of curriculum in standard settings. Despite curriculum could still influence non-convex problems [12], empirical results in the ML field are not showing clear signals of memory retention. A possible explanation is that relying on memory effects in the learning dynamics would require one to hit a sweet spot in the learning rate value and in the number of training epochs, and this seems hard to be achieved consistently. These observations raise the theoretical question of how curriculum learning could induce a non-vanishing effect in batch learning settings.

To instantiate a memory effect in our model, we propose biasing the optimisation landscape via a Gaussian prior, centered around the optimiser of the previous learning phase. The additional term in the loss acts as an elastic coupling between the successive phases, and the associated intensity *γ*_12_ is then an additional hyper-parameter of the model. This scheme is similar to regularisation methods proposed against catastrophic interference in continual learning, such as synaptic intelligence [39].

Tools from statistical physics can be used to analytically compute test performance under this scheme. In order to simplify the presentation, we first consider just two learning phases. It is natural to frame this setting as a two-level problem, involving two systems with independent copies of the network weights ***W***_1_ and ***W***_2_. In a typical statistical physics approach, we associate a Boltzmann–Gibbs measure to the systems, with an energy function determined by the regularised logistic loss ${\mathcal{L}}_{\gamma }$. While the statistical properties of the first system can be determined self-consistently, the added elastic interaction creates a dependence of the second measure on the configurations of the first system. In mathematical terms, the coupled system is represented by the following partition function:

\begin{align*}\hfill {\langle Z({\boldsymbol{W}}_{2},{\boldsymbol{W}}_{1};{\mathcal{D}}_{1},{\mathcal{D}}_{2})\rangle }_{{\boldsymbol{W}}_{1}}&amp; =\int \mathrm{d}{\boldsymbol{W}}_{1}\frac{{\text{e}}^{-{\beta }_{1}{\mathcal{L}}_{{\gamma }_{1}}({\boldsymbol{W}}_{1},{\mathcal{D}}_{1})}}{{Z}_{1}({\boldsymbol{W}}_{1})}\mathrm{log}\hfill \\ \hfill &amp; \quad \times \int \mathrm{d}{\boldsymbol{W}}_{2}\,{\text{e}}^{-{\beta }_{2}\left({\mathcal{L}}_{{\gamma }_{2}}({\boldsymbol{W}}_{2},{\mathcal{D}}_{2})+\frac{{\gamma }_{12}}{2}{\Vert}{\boldsymbol{W}}_{2}-{\boldsymbol{W}}_{1}{{\Vert}}_{2}^{2}\right)}\hfill \end{align*}

where ${\mathcal{D}}_{1},{\mathcal{D}}_{2}$ denote the two dataset slices. This object represents the normalisation of the Boltzmann–Gibbs measure, and allows one to extract relevant information on the asymptotic behaviour of our model. The optimisations entailed in each learning phase can be described in the ‘low noise’ limit of *β*_1_, *β*_2_ → ∞, where the measures focus on the minimisers of the respective losses. In order to study a self-averaging quantity that does not depend on a specific realisation of the dataset, we aim to compute the associated average free-energy:

\begin{equation*}{\Phi}=\underset{N\to \infty }{\mathrm{lim}}\underset{{\beta }_{1},{\beta }_{2}\to \infty }{\mathrm{lim}}\frac{1}{{\beta }_{2}N}{\left\langle \mathrm{log}{\left\langle Z({\boldsymbol{W}}_{2},{\boldsymbol{W}}_{1};{\mathcal{D}}_{1},{\mathcal{D}}_{2})\right\rangle }_{{\boldsymbol{W}}_{1}}\right\rangle }_{{\mathcal{D}}_{1},{\mathcal{D}}_{2}}.\end{equation*}

This quantity can be seen as a special case of the so-called Franz–Parisi potential computation [40, 41], and the entailed double average can be evaluated through the replica method. Refer to the appendices for details.

**Figure 4.**
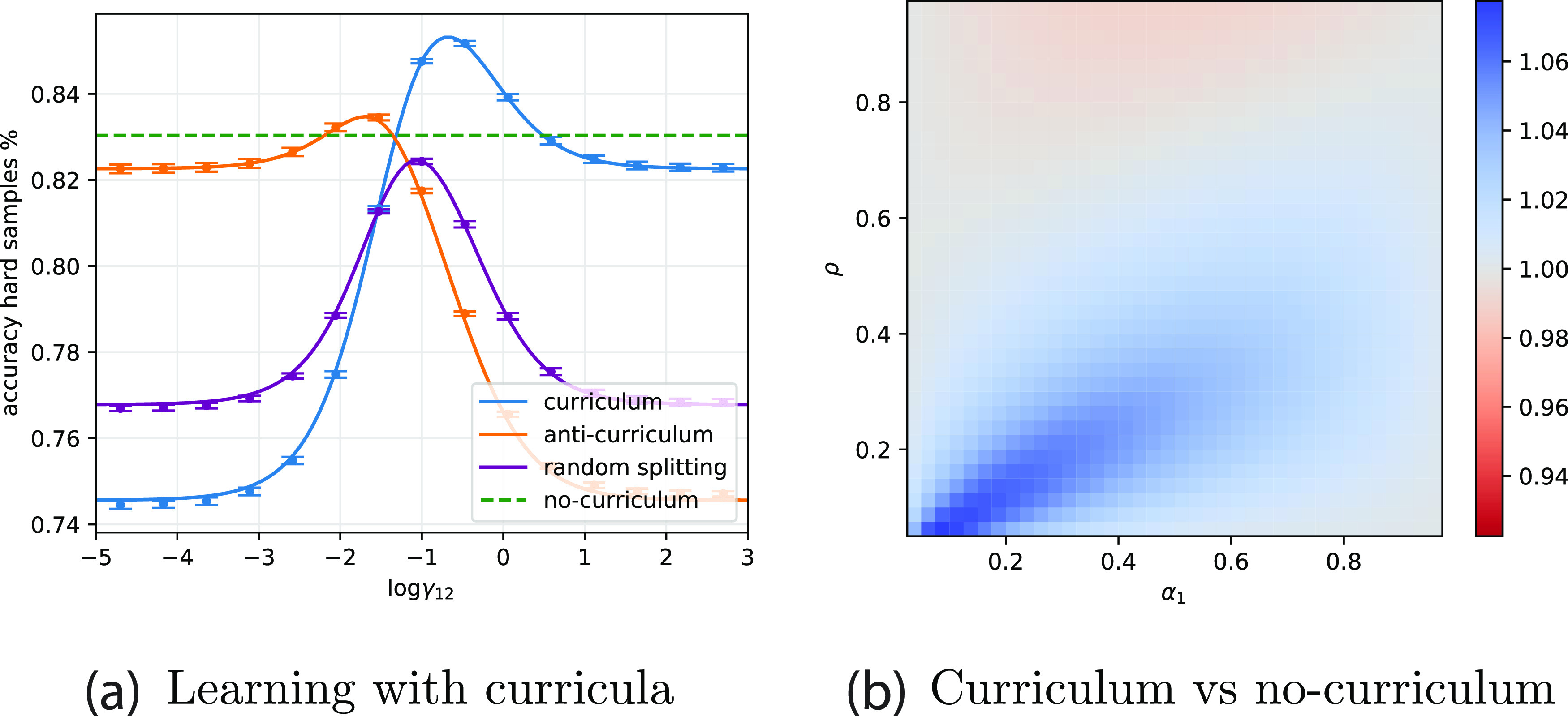
Effect of elastic coupling (Gaussian prior) between curriculum phases. (a) Comparison between asymptotic performance of curricula (full lines) and single batch learning, at *α*_1_ = 1, *α*_2_ = 1, with a regularisation *γ*_1_ that yields the best generalisation when learning the entire dataset (in principle not optimal for the other strategies). The points represent the results from 10 numerical simulations at size *N* = 2000. Parameters: *ρ* = 0.50, Δ_1_ = 0 and Δ_2_ = 1. (b) ratio between the accuracy reached by curriculum learning over anti-curriculum as a function of the number of easy samples in a dataset of dimension *α*_1_ + *α*_2_ = 1, and of the sparsity level of the teacher *ρ*. Note that *ρ* can also be seen as the fraction of relevant components in the inputs. Δ_1_ = 0 and Δ_1_ = 1. *γ*_1_ = *γ*_2_ and *γ*_12_ where set the values that optimise test performance.

Similar to the online case, in high-dimensions the free-entropy concentrates on a deterministic function that depends on several order parameters that capture the geometrical distribution of teacher and student configurations. In addition to those already introduced in section 3, we also have *δQ*, which is linked to the variance of the student norm. Moreover, for each order parameter we also need to introduce a conjugate parameter, denoted in the following with the hat symbol. The final expression for the free-energy reads:

\begin{align*}\hfill {\Phi}&amp; =\mathrm{e}\mathrm{x}\mathrm{t}\mathrm{r}\left[-\left(\hat{R}R+\frac{1}{2}\left({\left(\hat{Q}\delta Q-\delta \hat{Q}Q\right)}_{r+i}\right)\right)+\,{g}_{S}({\gamma }_{1},{\gamma }_{2},{\gamma }_{12})\right.\hfill \\ \hfill &amp; \quad \left.+{\alpha }_{1}\,{g}_{E}\left({{\Delta}}_{1}\right)+{\alpha }_{2}\,{g}_{E}\left({{\Delta}}_{2}\right)\right]\hfill \end{align*}

where *g*_*S*_ and *g*_*E*_ are two scalar functions, often called entropic and energetic channels, that encode the dependence of the optimisation problem on the Gaussian prior and the logistic loss respectively. The extremum condition for the free-energy yields a system of fixed-point equations that converge to an asymptotic prediction for the order parameters, comparable with the results of numerical simulations on large instances, figure 4. At convergence, the order parameters can be inserted again in equation (2) to obtain an estimate of the test accuracy. Note that this formalism is not limited to two phases, but can be extended to the case of a discrete number of sequential stages.

*The importance of sparsity*. Sparsity is a key ingredient in determining the impact of curriculum strategies. It naturally introduces a notion of relevant and irrelevant input components, and defines a secondary learning goal, i.e. identifying what part of the presented data should be disregarded by the model. Curriculum learning can be extremely helpful in this identification process, since the easy samples are more transparent to this structure. This is also observed in human experiments [6]. However, the relative difficulty of the problem of inferring the support of the teacher and the problem of aligning with its non-zero components depends on the degree of sparsity *ρ*, so the effectiveness of curriculum can vary with it.

In the right panel of figure 4, we explore the interplay between the sparsity of the teacher *ρ* and the fraction of easy samples in the dataset *α*_1_, comparing curriculum with the no-curriculum baseline. The phase diagram highlights the variability in the impact of the curriculum ordering:

• Curriculum is most effective at low values of *ρ* and close to the diagonal, where the fraction of easy examples in the dataset is comparable to the fraction of relevant dimensions.
• When *ρ* > 0.5, the possible gain from ordering the samples according to difficulty is counterbalanced by the instrinsic cost of splitting the information content into two blocks, thus curriculum can become detrimental.
• When *α*_1_ is too small compared to *ρ* (above diagonal), the first stage in the curriculum strategy can only help in the support identification problem, but will not allow a good estimation of the direction of the teacher. Because of the elastic prior, the second stage cannot improve too much over it and the effect of curriculum is small.
• When *α* is larger than the sparsity (below diagonal), the easy examples contain sufficient information for solving both the support and the teacher estimation problems, and this information is also exploited by the baseline. Thus the improvement of curriculum becomes negligible.

We refer to the appendices for an in-depth comparison with anti-curriculum.

**Figure 5.**
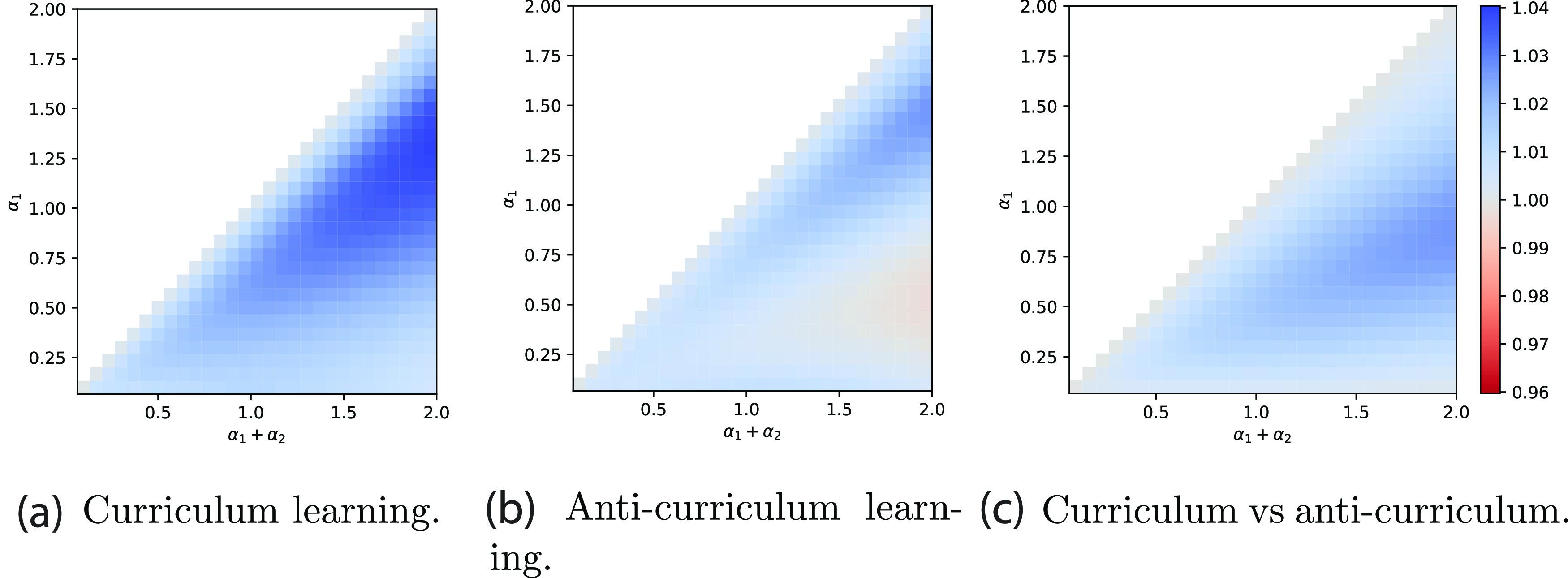
Phase diagram for the performance gap in the batch setting. The colour scale shows the ratio of the accuracy on hard instances for curriculum over no-curriculum (a), anti-curriculum over no-curriculum (b), and curriculum over anti-curriculum (c), as a function of the total dataset size (*α*_1_ + *α*_2_) and easy dataset size (*α*_1_). In contrast to the online case, performance benefits are greater and curriculum is strictly better than anti-curriculum. Both *γ*_1_ = *γ*_2_ and *γ*_12_ are optimised point-wise, in order to yield the best test accuracy. Parameters: *ρ* = 0.50, Δ_1_ = 0, Δ_2_ = 1.

*Asymptotic advantages of curriculum*. Contrary to the online SGD case, if the fraction of relevant directions is small, batch learning with elastic coupling notably improves test accuracy of both curriculum and anti-curriculum above the baseline. This confirms the utility of curriculum strategies when the signal is partially ‘hidden in clutter’ [42]. Figure 5 shows similar phase diagrams to figure 2 but for the batch setting. At each point in the phase diagram the regularisation level *γ*_1_ = *γ*_2_ and the coupling *γ*_12_ are optimised to yield the best accuracy. In batch learning the performance order appears to be nearly always preserved: curriculum followed by anti-curriculum followed by baseline. We remark that this result is not trivial as splitting the learning process in two stages is not advantageous per se. Note that in the appendices we observe similar improvement applying the elastic coupling on the online setting and on real data.

## 5. Connection with experimental literature

Recent work has suggested that curriculum learning could provide an important window into the learning algorithms at work in biology [44]. Our analysis makes several predictions for curriculum effects. In this section we assess these predictions based on connections to extant experiments and propose future experimental tests.

First, we find that a curriculum strategy yields a speed up in learning in all the tested settings (see figure 1(c)). This acceleration is broadly consistent with the findings from cognitive science [1, 2, 6]. By contrast, our results show that the speed improvement does not necessarily translate into a sizeable generalisation error improvement, and the performance achieved at the end of training can even deteriorate when learning hyperparameters are not fully optimised (cf figure 3). Deterioration due to curricula has generally not been reported in the psychology literature, though it has been observed in ML [15]. This fact may suggest that animals naturally learn with near-optimal hyperparameters such that curricula generally confer benefits.

**Figure 6.**
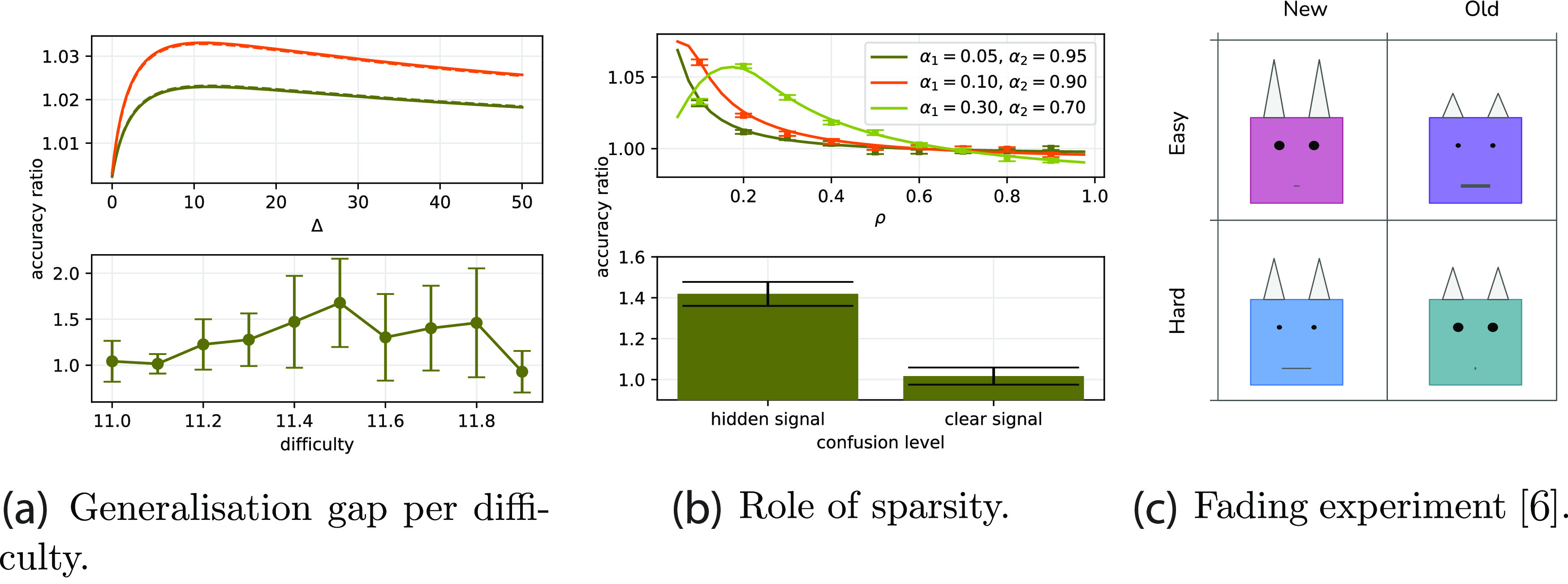
Connection with psychology experiments. (a) (Top) Accuracy ratio of different strategies in the model, with curriculum/no-curriculum in green and curriculum/anti-curriculum in orange. The ratio shows non-monotonic behaviour. (Bottom) The accuracy ratio obtained by [43]. Parameters *ρ* = 0.5, Δ_1_ = 0.0, Δ_2_ = 1.0, *α*_1_ = 1, *α*_2_ = 1 and optimal learning rate, variance at initialisation and weight decay. (b) (Top) Dependence on the sparsity of the generalisation gain of curriculum over no-curriculum, measured as ratio between final accuracy, for fixed total dataset size (*α*_1_ + *α*_2_ = 1). (Bottom) The ratio obtained from experiments 3 and 4 of [6]. (c) Example cartoon stimuli from the ‘fading’ paradigm used in [6], where participants distinguish daemons of the old world from daemons of the new world. The distinguishing feature (horn length) is diluted among many irrelevant features (colour, eye size, mouth size). Highlighting the relevant feature to participants leads to better and faster learning.

A more specific observation concerns the performance on different difficulties after learning. As reported in [43], human and rodent subjects trained in an auditory task using curricula showed the greatest improvement for intermediate level of difficulties as depicted in figure 6(a) (bottom). The same conclusion can be drawn from the experiment of [7, 8], where, surprisingly, subjects trained with curricula to classify medical images showed poor performance in hard tasks compared to the control group. To address this phenomenon, we calculate accuracy as a function of difficulty in the model in figure 6(a) (top). Consistent with these experiments, we find regimes where the gap between curriculum learning and the baseline is non-monotonic, with the largest performance gain for intermediate difficulties. Contrary to [7, 8], however, we do not observe negative effects of curriculum for high difficulties. Further experiments that more systematically manipulate training and transfer difficulties could provide a stronger test of these predictions.

A key ingredient in our model is the role of sparsity, such that a small signal is embedded amidst many irrelevant features. Experimentally, the importance of having many factors of variation to obtaining a curriculum effect has been documented in the ‘fading’ experiments of [6]. Human subjects were trained on classification tasks involving stimuli with one task-relevant feature dimension and a variable number of task-irrelevant feature dimensions. Example cartoon ‘daemon’ stimuli are depicted in figure 6(c), where for instance horn height might be the distinguishing feature while colour, eye size, and mouth size might constitute task-irrelevant features. Without any irrelevant factors of variation (*ρ* = 1), they report no curriculum benefit. By contrast when 75% of features are irrelevant (*ρ* = 0.25), they record a strong curriculum effect, as shown in figure 6(b) bottom. This qualitative trend is also observed in our model (figure 6(b), top). While these experiments tested only two sparsity levels, further experiments could sample this dimension more extensively and test for interactions with the fraction of easy and hard examples. We note that while the connectionist literature has addressed the effect of curriculum in several settings [10, 11, 45, 46], we found that easy-to-hard effects appear even in a simple setup without need for complex networks and/or dynamics.

**Figure 7.**
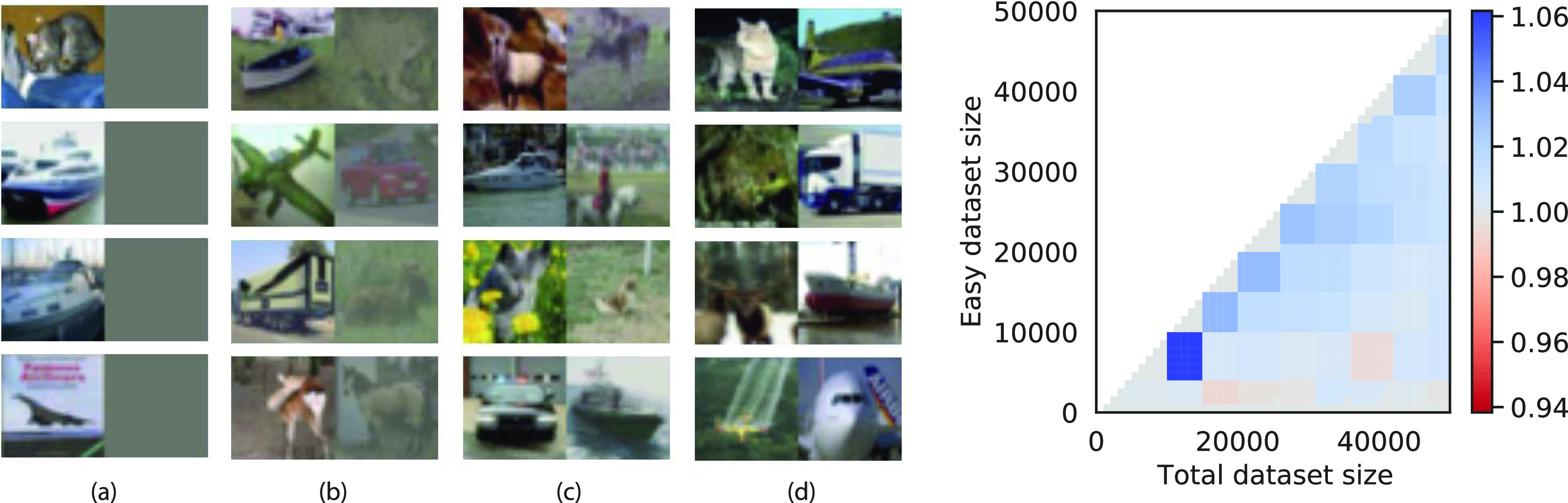
Experimental setting on CIFAR10-derived data. (a) Input samples combine a task-relevant image with a distractor image, and become progressively harder from left to right. (b) Ratio between final accuracy on hard instances for curriculum learning versus no curriculum. *η*, *γ*, *γ*_12_, init, and stopping time are optimised.

Finally, our results may shed light on self-generated curricula during human development [47, 48]. Children undergo a spurt of vocabulary development that coincides with their ability to grasp and centre objects in the visual field [48]. Quantitative estimates of the amount of clutter (irrelevant objects) in self-generated views decrease due to this grasping ability, yielding a self-generated curriculum [42, 49]. Our model similarly predicts that reducing clutter should improve learning speed and performance. To verify this in a richer visual setting, we apply our curriculum-aware algorithm on real data where the loss is modified to keep track of the different phases of learning. We construct a simple cluttered object recognition task from the CIFAR10 dataset [50] by patching two images together into a 32 × 64 input image (figure 7(a)). The network has to learn that the classification depends only on the left image, while the right image is a distractor that is irrelevant to the classification. Easy and hard instances are obtained by reducing the brightness of the distractor. We train a single-layer network with the cross-entropy loss and the curriculum protocol with Gaussian prior between the two stages. Weights are optimised using SGD and momentum, with an annealed learning rate. All training parameters are optimised, and full details are presented in the appendices. Figure 7 shows a robust curriculum advantage in this setting, suggesting a possible functional benefit for children’s self-generated curriculum.

## 6. Conclusions

We analysed a model of curriculum learning introduced by [12] and amenable of analytical treatment. This simple setting sheds light on results observed in the cognitive science and ML literature, and the theoretical tractability allows for exploration of a wide range of parameters that would be costly to obtain through experiments. Future work will need to move beyond models with simple loss landscapes to address the impact of curricula in complex tasks like reinforcement learning. Nevertheless, the model recapitulates a variety of observations in the literature [43, 51, 52], revealing that easy-to-hard effects can appear when a sparse signal is embedded in many irrelevant dimensions of variation. We find that making the algorithm curriculum-aware by modifying the loss can better exploit curricula, offering a potential route for improved practical algorithms. Other curriculum-aware approaches are possible such as adapting the learning algorithm [53] or the architecture [10]. On the psychology side, some of our predictions can help in designing new experiments. The benefit of anti-curriculum learning for intermediate sparsity, is a counter-intuitive result testable in animal experiments.

## Figures and Tables

**Figure A.1.**
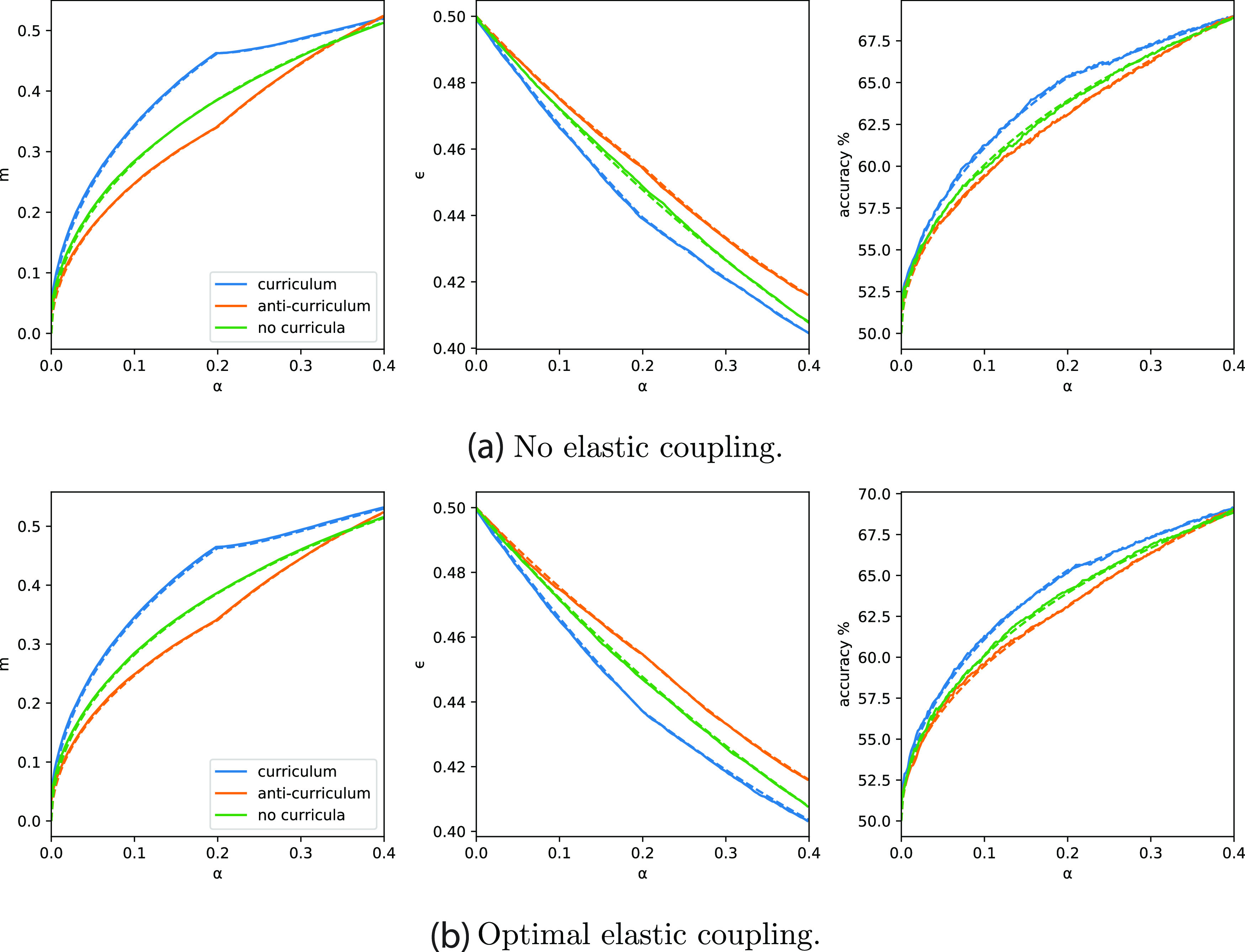
Effect of elastic coupling in the curriculum. Figures showing the teacher–student cosine, the validation loss, and the accuracy of the three learning strategies. The two figures show the performance in presence (above) and absence (below) of elastic coupling. The dashed lines are obtained from the theoretical analysis, the full line come from the average of 500 simulations. The parameters *η*, *γ*, initialisation are set to the optimal values for each protocol. Parameters: *ρ* = 0.5, *α*_1_ = 0.2, *α*_2_ = 0.2, Δ_1_ = 0, Δ_2_ = 1.

**Figure C.1.**
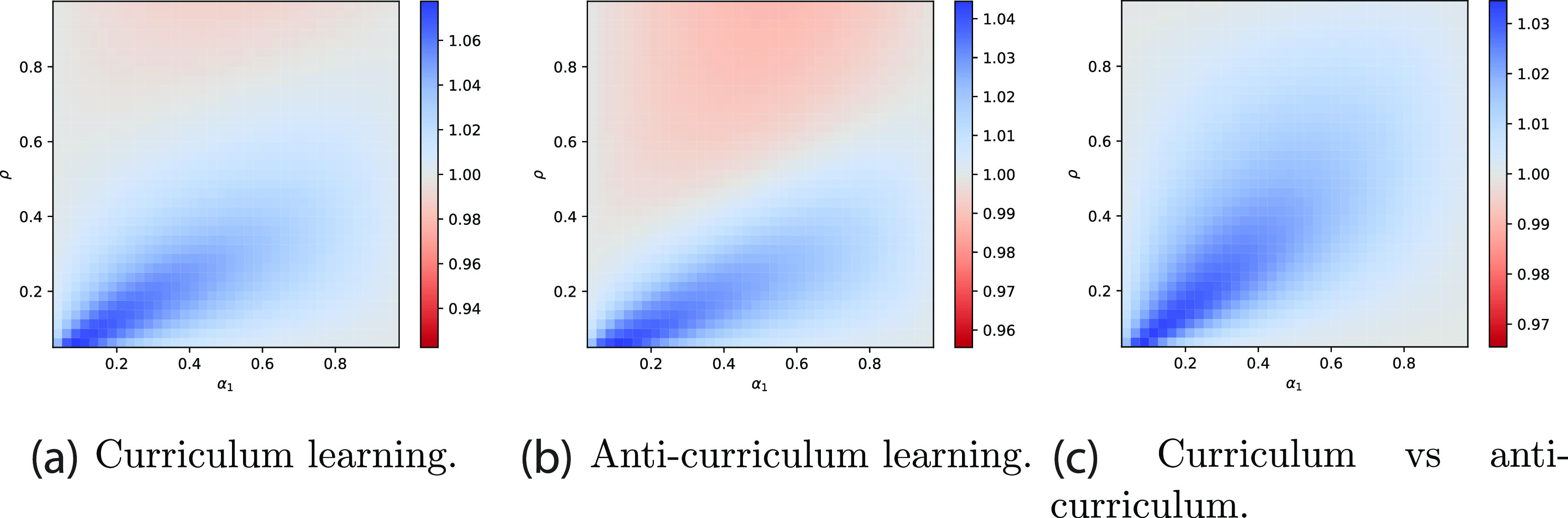
Effect of sparsity. Phase diagram on the effect of sparsity, figure 4(b), extended for all learning protocols.

## Appendix A. State evolution of the online dynamics

In this section we show how to derive the dynamical equations for the online dynamics. The equations given in an implicit form in the main text, ${f}_{{Q}_{r}},{f}_{{Q}_{i}},{f}_{R}$, are reported explicitly at the end of the next section, equations (A.22)–(A.24). Finally, in the subsequent section, we comment on how the state evolution is modified to deal with the Gaussian priors and we derive the new dynamical equations for that case.

*Derivation*. We follow the derivation proposed in [26, 35] to derive the averaged high-dimensional dynamical equations. The student is a one-layer network that minimises sample-wise the square error

\begin{equation*}{\mathcal{L}}^{\mu }=\frac{1}{2}{\left({y}^{\mu }-{\hat{y}}^{\mu }\right)}^{2}\doteq \frac{1}{2}{\left({\delta }^{\mu }\right)}^{2}.\end{equation*}

Given $\phi (\cdot )=\mathrm{sign}(\cdot ),\sigma (\cdot )=\mathrm{e}\mathrm{r}\mathrm{f}(\cdot /\sqrt{2})$, the online stochastic gradient descent updates are

\begin{equation*}{\boldsymbol{W}}^{\mu +1}={\boldsymbol{W}}^{\mu }-\frac{\eta }{\sqrt{N}}{\sigma }^{\prime }({\lambda }_{r}^{\mu }+{\lambda }_{i}^{\mu }){\delta }^{\mu }{\boldsymbol{x}}^{\mu },\end{equation*}

with

\begin{equation*}{\lambda }_{r}^{\mu }=\frac{1}{\sqrt{N}}{\boldsymbol{W}}_{r}\cdot {\boldsymbol{x}}_{r}^{\mu },\end{equation*}

\begin{equation*}{\lambda }_{i}^{\mu }=\frac{1}{\sqrt{N}}{\boldsymbol{W}}_{i}\cdot {\boldsymbol{x}}_{i}^{\mu },\end{equation*}

\begin{equation*}{\rho }^{\mu }=\frac{1}{\sqrt{N}}{\boldsymbol{W}}_{T}\cdot {\boldsymbol{x}}_{r}^{\mu }.\end{equation*}

The evolution of the dynamics can be tracked using four order parameters:

\begin{equation*}{Q}_{r}=\frac{1}{N}{\boldsymbol{W}}_{r}\cdot {\boldsymbol{W}}_{r},\end{equation*}

\begin{equation*}{Q}_{i}=\frac{1}{N}{\boldsymbol{W}}_{i}\cdot {\boldsymbol{W}}_{i},\end{equation*}

\begin{equation*}R=\frac{1}{N}{\boldsymbol{W}}_{r}\cdot {\boldsymbol{W}}_{T},\end{equation*}

\begin{equation*}T=\frac{1}{N}{\boldsymbol{W}}_{T}\cdot {\boldsymbol{W}}_{T};\end{equation*}

representing the overlaps between the weights of student (relevant and irrelevant parts) and teacher.

The evolution of those follow from the definition of the dynamics equation (A.2). In the high-dimensional limit the random variables in the problem concentrates around the mean, therefor to the leading order we have the following equations

\begin{equation*}{Q}_{r}[k+1]={Q}_{r}[k]+\frac{1}{N}\left[2\eta \mathbb{E}[\delta \ {\sigma }^{\prime }({\lambda }_{r}+{\lambda }_{i}){\lambda }_{r}]+\rho {\Delta}{\eta }^{2}\mathbb{E}[{\delta }^{2}\ {\sigma }^{\prime }{({\lambda }_{r}+{\lambda }_{i})}^{2}]\right];\end{equation*}

\begin{equation*}{Q}_{i}[k+1]={Q}_{r}[k]+\frac{1}{N}\left[2\eta \mathbb{E}[\delta \ {\sigma }^{\prime }({\lambda }_{r}+{\lambda }_{i}){\lambda }_{i}]+(1-\rho ){\Delta}{\eta }^{2}\mathbb{E}[{\delta }^{2}\ {\sigma }^{\prime }{({\lambda }_{r}+{\lambda }_{i})}^{2}]\right];\end{equation*}

\begin{equation*}\,T[k+1]={Q}_{r}[k]+\frac{1}{N}\left[\eta \mathbb{E}[\delta \ {\sigma }^{\prime }({\lambda }_{r}+{\lambda }_{i})\rho ]\right],\end{equation*}

where the expectation acts with respect to all the stochastic variables. In order to obtain explicit formulae we need to evaluate those averages. The random variables in the equations—*λ*_*r*_, *λ*_*i*_ and *ρ*—are Gaussian with zero mean, to characterise them we only need their covariance:

\begin{equation*}{{\Sigma}}_{{\lambda }_{r},{\lambda }_{i},\rho }=\left(\begin{matrix}\hfill {Q}_{r}\hfill &amp; \hfill 0\hfill &amp; \hfill R\hfill \\ \hfill 0\hfill &amp; \hfill {Q}_{i}\hfill &amp; \hfill 0\hfill \\ \hfill R\hfill &amp; \hfill 0\hfill &amp; \hfill T\hfill \end{matrix}\right).\end{equation*}

In order to derive analytical expression we must evaluate the expected values: $\mathbb{E}[\phi (\rho ){\sigma }^{\prime }(\lambda )\rho ]$, $\mathbb{E}[\phi (\rho ){\sigma }^{\prime }(\lambda )\lambda ]$, $\mathbb{E}[\sigma (\lambda ){\sigma }^{\prime }(\lambda )\rho ]$, $\mathbb{E}[\sigma (\lambda ){\sigma }^{\prime }(\lambda )\lambda ]$, $\mathbb{E}[\phi {(\rho )}^{2}{\sigma }^{\prime }{(\lambda )}^{2}]$, $\mathbb{E}[\sigma {(\lambda )}^{2}{\sigma }^{\prime }{(\lambda )}^{2}]$, and $\mathbb{E}[\phi (\rho )\sigma (\lambda ){\sigma }^{\prime }{(\lambda )}^{2}]$, where *σ* is the activation function of the student and *ϕ* is the activation function of the teacher (in particular *ϕ*(⋅) = sign(⋅) for classification).

\begin{equation*}\ \mathbb{E}[\phi (\rho ){\sigma }^{\prime }(\lambda )\rho ]=\frac{2}{\pi }\frac{\sqrt{T({Q}_{r}+{Q}_{i}+1)-{R}^{2}}}{{Q}_{r}+{Q}_{i}+1}\end{equation*}

\begin{equation*}\mathbb{E}[\phi (\rho ){\sigma }^{\prime }(\lambda ){\lambda }_{r}]=\frac{2}{\pi }\frac{R({Q}_{i}+1)}{{Q}_{r}+{Q}_{i}+1}\frac{1}{\sqrt{T({Q}_{r}+{Q}_{i}+1)+{R}^{2}}}.\end{equation*}

\begin{equation*}\mathbb{E}[\phi (\rho ){\sigma }^{\prime }(\lambda ){\lambda }_{i}]=-\frac{2}{\pi }\frac{R{Q}_{i}}{{Q}_{r}+{Q}_{i}+1}\frac{1}{\sqrt{T({Q}_{r}+{Q}_{i}+1)+{R}^{2}}}.\end{equation*}

\begin{equation*}\,\mathbb{E}[\sigma (\lambda ){\sigma }^{\prime }(\lambda )\rho ]=\frac{2}{\pi }\frac{R}{{Q}_{r}+{Q}_{i}+1}\sqrt{\frac{{Q}_{i}+1}{2{Q}_{i}^{2}+2{Q}_{r}{Q}_{i}+3{Q}_{i}+2{Q}_{r}+1}}.\end{equation*}

\begin{equation*}\mathbb{E}[\sigma (\lambda ){\sigma }^{\prime }(\lambda ){\lambda }_{r}]=\frac{2}{\pi }\frac{{Q}_{r}}{{Q}_{r}+{Q}_{i}+1}\sqrt{\frac{{Q}_{i}+1}{2{Q}_{i}^{2}+2{Q}_{r}{Q}_{i}+3{Q}_{i}+2{Q}_{r}+1}}.\end{equation*}

\begin{equation*}\mathbb{E}[\sigma (\lambda ){\sigma }^{\prime }(\lambda ){\lambda }_{i}]=\frac{2}{\pi }\frac{{Q}_{i}}{{Q}_{r}+{Q}_{i}+1}\sqrt{\frac{{Q}_{r}+1}{2{Q}_{r}^{2}+2{Q}_{r}{Q}_{i}+3{Q}_{r}+2{Q}_{i}+1}}.\end{equation*}

\begin{equation*}\,\mathbb{E}[\phi {(\rho )}^{2}{\sigma }^{\prime }{(\lambda )}^{2}]=\frac{2}{\pi }\frac{1}{\sqrt{2{Q}_{r}+2{Q}_{i}+1}}.\end{equation*}

\begin{equation*}\mathbb{E}[\sigma {(\lambda )}^{2}{\sigma }^{\prime }{(\lambda )}^{2}]=\frac{4}{{\pi }^{2}}\frac{1}{\sqrt{1+2({Q}_{r}+{Q}_{i})}}{\sin }^{-1}\left(\frac{{Q}_{r}+{Q}_{i}}{1+3({Q}_{r}+{Q}_{i})}\right).\end{equation*}

\begin{align*}\hfill &amp; \mathbb{E}[\phi (\rho )\sigma (\lambda ){\sigma }^{\prime }{(\lambda )}^{2}]=\frac{4}{{\pi }^{2}}\frac{1}{\sqrt{2({Q}_{r}+{Q}_{i})+1}}\hfill \\ \hfill &amp; \quad \hspace{25.0pt}\times {\sin }^{-1}\left(\frac{R\sqrt{{Q}_{r}+{Q}_{i}}}{\sqrt{3({Q}_{r}+{Q}_{i})+1}\sqrt{(2{Q}_{r}+2{Q}_{i}+1)[T({Q}_{r}+{Q}_{i})-{R}^{2}]+{R}^{2}}}\right).\hfill \end{align*}

Finally, we can substitute those equations into the equations (A.10)–(A.12) and obtained the state evolution equations used in the main section 3:

\begin{align*}\hfill &amp; {f}_{{Q}_{r}}\left({Q}_{r}[k],{Q}_{i}[k],R[k],T\right)\,={(1-\eta \gamma )}^{2}{Q}_{r}[k]+\frac{4\eta (1-\eta \gamma )}{N\pi ({Q}_{r}[k]+{\Delta}{Q}_{i}[k]+1)}\hfill \\ \hfill &amp; \hspace{25.0pt}\quad \times \left[\frac{R[k]({\Delta}{Q}_{i}[k]+1)}{\sqrt{T({Q}_{r}[k]+{\Delta}{Q}_{i}[k]+1)+R{[k]}^{2}}}-\frac{{Q}_{r}[k]}{\sqrt{2{Q}_{r}[k]+2{\Delta}{Q}_{i}[k]+1}}\right]\hfill \\ \hfill &amp; \hspace{25.0pt}\quad +\frac{4}{{\pi }^{2}}\frac{\rho {\eta }^{2}}{N\sqrt{2({Q}_{r}[k]+{\Delta}{Q}_{i}[k])+1}}\left[\frac{\pi }{2}+{\sin }^{-1}\left(\frac{{Q}_{r}[k]+{\Delta}{Q}_{i}[k]}{1+3({Q}_{r}[k]+{\Delta}{Q}_{i}[k])}\right)+\right.\hfill \\ \hfill &amp; \hspace{25.0pt}\quad \left.-2\enspace {\sin }^{-1}\left(\frac{R[k]}{\sqrt{3({Q}_{r}[k]+{\Delta}{Q}_{i}[k])+1}\sqrt{T(2{Q}_{r}[k]+2{\Delta}{Q}_{i}[k]+1)-2R{[k]}^{2}}}\right)\right];\hfill \end{align*}

\begin{align*}\hfill &amp; {f}_{{Q}_{i}}\left({Q}_{r}[k],{Q}_{i}[k],R[k],T\right)\,={(1-\eta \gamma )}^{2}{Q}_{i}[k]-\frac{4\eta (1-\eta \gamma ){\Delta}{Q}_{i}[k]}{N\pi ({Q}_{r}[k]+{\Delta}{Q}_{i}[k]+1)}\hfill \\ \hfill &amp; \hspace{25.0pt}\quad \times \left[\frac{R[k]}{\sqrt{T({Q}_{r}[k]+{\Delta}{Q}_{i}[k]+1)+R{[k]}^{2}}}+\frac{1}{\sqrt{2{Q}_{r}[k]+2{\Delta}{Q}_{i}[k]+1}}\right]\hfill \\ \hfill &amp; \hspace{25.0pt}\quad +\frac{4}{{\pi }^{2}}\frac{(1-\rho ){\Delta}{\eta }^{2}}{N\sqrt{2({Q}_{r}[k]+{\Delta}{Q}_{i}[k])+1}}\left[\frac{\pi }{2}+{\sin }^{-1}\left(\frac{{Q}_{r}[k]+{\Delta}{Q}_{i}[k]}{1+3({Q}_{r}[k]+{\Delta}{Q}_{i}[k])}\right)+\right.\hfill \\ \hfill &amp; \hspace{25.0pt}\quad \left.-2\enspace {\sin }^{-1}\left(\frac{R[k]}{\sqrt{3({Q}_{r}[k]+{\Delta}{Q}_{i}[k])+1}\sqrt{T(2{Q}_{r}[k]+2{\Delta}{Q}_{i}[k]+1)-2R{[k]}^{2}}}\right)\right];\hfill \end{align*}

\begin{align*}\hfill &amp; {f}_{R}\left({Q}_{r}[k],{Q}_{i}[k],R[k],T\right)=(1-\eta \gamma )R[k]+\frac{2\eta }{N\pi ({Q}_{r}[k]+{\Delta}{Q}_{i}[k]+1)}\hfill \\ \hfill &amp; \quad \hspace{25.0pt}\times \left[\frac{T({Q}_{r}[k]+{\Delta}{Q}_{i}[k]+1)-R{[k]}^{2}}{\sqrt{T({Q}_{r}[k]+{\Delta}{Q}_{i}[k]+1)-R{[k]}^{2}}}-\frac{R[k]}{\sqrt{2{Q}_{r}[k]+2{\Delta}{Q}_{i}[k]+1}}\right].\hfill \end{align*}

*Elastic coupling*. The introduction of the elastic coupling between stages of learning adds five new order parameters: three of them are just reminder of the previous stage and do not need to by updated ${\tilde{Q}}_{r}={\boldsymbol{W}}_{1}^{\,r}\cdot {\boldsymbol{W}}_{1}^{\,r}/N$, ${\tilde{Q}}_{i}={\boldsymbol{W}}_{1}^{\,i}\cdot {\boldsymbol{W}}_{1}^{\,i}/N$, and $\tilde{R}={\boldsymbol{W}}_{1}^{\,i}\cdot {\boldsymbol{W}}^{\,T}/N$; two measure the correlation between the two stages ${S}_{r}={\boldsymbol{W}}_{1}^{\,r}\cdot {\boldsymbol{W}}_{2}^{\,r}/N$ and ${S}_{i}={\boldsymbol{W}}_{1}^{\,i}\cdot {\boldsymbol{W}}_{2}^{\,i}/N$ to the equations. These terms have associated their own state evolution equations slightly modified the updates of the other order parameters.

\begin{align*}\hfill {Q}_{r}[k+1]&amp; ={(1-\eta \gamma +\eta {\gamma }_{12})}^{2}{Q}_{r}[k]+\frac{2\eta }{N}(1-\eta \gamma +\eta {\gamma }_{12})\mathbb{E}[\delta \ {\sigma }^{\prime }({\lambda }_{r}+{\lambda }_{i}){\lambda }_{r}]\hfill \\ \hfill &amp; \quad +\rho {\Delta}\frac{{\eta }^{2}}{N}\mathbb{E}[{\delta }^{2}\ {\sigma }^{\prime }{({\lambda }_{r}+{\lambda }_{i})}^{2}]+2\eta {\gamma }_{12}(1-\eta \gamma +\eta {\gamma }_{12}){S}_{r}[k]+{\eta }^{2}{\gamma }_{12}^{2}{\tilde{Q}}_{r}[k]\hfill \\ \hfill &amp; \quad -\frac{2{\eta }^{2}{\gamma }_{12}}{N}\mathbb{E}[\delta \ {\sigma }^{\prime }({\lambda }_{r}+{\lambda }_{i}){\tilde{\lambda }}_{r}];\hfill \end{align*}

\begin{align*}\hfill {Q}_{i}[k+1]&amp; ={(1-\eta \gamma +\eta {\gamma }_{12})}^{2}{Q}_{i}[k]+\frac{2\eta }{N}(1-\eta \gamma +\eta {\gamma }_{12})\mathbb{E}[\delta \ {\sigma }^{\prime }({\lambda }_{r}+{\lambda }_{i}){\lambda }_{i}]\hfill \\ \hfill &amp; \quad +(1-\rho ){\Delta}\frac{{\eta }^{2}}{N}\mathbb{E}[{\delta }^{2}\ {\sigma }^{\prime }{({\lambda }_{r}+{\lambda }_{i})}^{2}]+2\eta {\gamma }_{12}(1-\eta \gamma +\eta {\gamma }_{12}){S}_{i}[k]\hfill \\ \hfill &amp; \quad +{\eta }^{2}{\gamma }_{12}^{2}{\tilde{Q}}_{i}[k]-\frac{2{\eta }^{2}{\gamma }_{12}}{N}\mathbb{E}[\delta \ {\sigma }^{\prime }({\lambda }_{r}+{\lambda }_{i}){\tilde{\lambda }}_{i}];\hfill \end{align*}

\begin{equation*}R[k+1]=(1-\eta \gamma +\eta {\gamma }_{12})R[k]+\frac{\eta }{N}\mathbb{E}[\delta \ {\sigma }^{\prime }({\lambda }_{r}+{\lambda }_{i})\rho ]-\eta {\gamma }_{12}\tilde{R}[k];\end{equation*}

\begin{equation*}{S}_{r}[k+1]=(1-\eta \gamma +\eta {\gamma }_{12}){S}_{r}[k]+\frac{\eta }{N}\mathbb{E}[\delta \ {\sigma }^{\prime }({\lambda }_{r}+{\lambda }_{i}){\tilde{\lambda }}_{r}]-\eta {\gamma }_{12}{\tilde{Q}}_{r}[k];\end{equation*}

\begin{equation*}{S}_{i}[k+1]=(1-\eta \gamma +\eta {\gamma }_{12}){S}_{i}[k]+\frac{\eta }{N}\mathbb{E}[\delta \ {\sigma }^{\prime }({\lambda }_{r}+{\lambda }_{i}){\tilde{\lambda }}_{i}]-\eta {\gamma }_{12}{\tilde{Q}}_{i}[k].\end{equation*}

Introduced ${\tilde{\lambda }}_{r}=\frac{1}{\sqrt{N}}{\boldsymbol{x}}_{r}\cdot {\tilde{\boldsymbol{W}}}_{r}$ and ${\tilde{\lambda }}_{i}=\frac{1}{\sqrt{N}}{\boldsymbol{x}}_{i}\cdot {\tilde{\boldsymbol{W}}}_{i}$, this two additional random variables need to be averaged together with the others. The joint distribution of ${\lambda }_{r},{\lambda }_{i},{\tilde{\lambda }}_{r},{\tilde{\lambda }}_{i},\rho $ is still Gaussian with zero mean and covariance

\begin{equation*}{{\Sigma}}_{{\lambda }_{r},{\lambda }_{i},{\tilde{\lambda }}_{r},{\tilde{\lambda }}_{i},\rho }=\left(\begin{matrix}\hfill {Q}_{r}\hfill &amp; \hfill 0\hfill &amp; \hfill {\tilde{S}}_{r}\hfill &amp; \hfill 0\hfill &amp; \hfill R\hfill \\ \hfill 0\hfill &amp; \hfill {Q}_{i}\hfill &amp; \hfill 0\hfill &amp; \hfill {\tilde{S}}_{i}\hfill &amp; \hfill 0\hfill \\ \hfill {\tilde{S}}_{r}\hfill &amp; \hfill 0\hfill &amp; \hfill {\tilde{Q}}_{r}\hfill &amp; \hfill 0\hfill &amp; \hfill \tilde{R}\hfill \\ \hfill 0\hfill &amp; \hfill {\tilde{S}}_{i}\hfill &amp; \hfill 0\hfill &amp; \hfill {\tilde{Q}}_{i}\hfill &amp; \hfill 0\hfill \\ \hfill R\hfill &amp; \hfill 0\hfill &amp; \hfill \tilde{R}\hfill &amp; \hfill 0\hfill &amp; \hfill T\hfill \end{matrix}\right).\end{equation*}

Notice that, a part from a slight change of the existing equations, the coupling introduces only two additional integrals $\mathbb{E}[\delta \ {\sigma }^{\prime }({\lambda }_{r}+{\lambda }_{i}){\tilde{\lambda }}_{r}]$ and $\mathbb{E}[\delta \ {\sigma }^{\prime }({\lambda }_{r}+{\lambda }_{i}){\tilde{\lambda }}_{i}]$. After long, but straightforward, computations we obtain

\begin{align*}\hfill \mathbb{E}[\delta \ {\sigma }^{\prime }({\lambda }_{r}+{\lambda }_{i}){\tilde{\lambda }}_{r}]&amp; =\frac{2}{\pi }\frac{{S}_{r}}{{Q}_{r}+{Q}_{i}+1}\frac{{Q}_{i}+1}{2{Q}_{i}^{2}+2{Q}_{r}{Q}_{i}+3{Q}_{i}+2{Q}_{r}+1}+\hfill \\ \hfill &amp; \quad -\frac{2}{\pi }\frac{T{S}_{r}-R\tilde{R}}{{Q}_{r}T-{R}^{2}}\frac{R({Q}_{i}+1)}{{Q}_{r}+{Q}_{i}+1}\frac{1}{\sqrt{T({Q}_{r}+{Q}_{i}+1)-{R}^{2}}}+\hfill \\ \hfill &amp; \quad -\frac{2}{\pi }\frac{T\tilde{R}-R{S}_{r}}{{Q}_{r}T-{R}^{2}}\frac{1}{\sqrt{T({Q}_{r}+{Q}_{i}+1)-{R}^{2}}}\hfill \\ \hfill &amp; \quad \times \frac{1}{\frac{1}{T}+\frac{{R}^{2}}{{Q}_{r}T-{R}^{2}}\left(\frac{1}{T}-\frac{{Q}_{i}+1}{T({Q}_{r}+{Q}_{i}+1)-{R}^{2}}\right)},\hfill \end{align*}

\begin{align*}\hfill \mathbb{E}[\delta \ {\sigma }^{\prime }({\lambda }_{r}+{\lambda }_{i}){\tilde{\lambda }}_{i}]&amp; =\frac{2}{\pi }\frac{{S}_{i}}{{Q}_{r}+{Q}_{i}+1}\frac{{Q}_{r}+1}{2{Q}_{r}^{2}+2{Q}_{r}{Q}_{i}+3{Q}_{r}+2{Q}_{i}+1}+\hfill \\ \hfill &amp; \quad -\frac{2}{\pi }\frac{{S}_{i}R}{{Q}_{r}+{Q}_{i}+1}\frac{1}{\sqrt{T({Q}_{r}+{Q}_{i}+1)-{R}^{2}}}.\hfill \end{align*}

Finally all the expected values are known and we can obtain the analytic updates equations (A.25)–(A.29) with the coupling. Figure A.1(a) shows an instance of the problem at *α*_1_ = 0.2 and *α*_2_ = 0.2, a situation that is particularly adversarial for curriculum according the phase diagram figure 2. This situation is treated by the introduction of Gaussian priors, figure A.1(b), consistently with the phase diagram in figure 7(c).

## Appendix B. Replica computation for the batch case

We here the detailed replica computation employed to obtain the analytic description of curriculum learning in the batch case, in section 4. As mentioned in the main, we aim to study a coupled system, represented by the following partition function:

\begin{align*}\hfill {\left\langle Z({\boldsymbol{W}}_{2},{\boldsymbol{W}}_{1};{\mathcal{D}}_{1},{\mathcal{D}}_{2})\right\rangle }_{{\boldsymbol{W}}_{1}}&amp; =\int \mathrm{d}{\boldsymbol{W}}_{1}\frac{{\text{e}}^{-{\beta }_{1}{\mathcal{L}}_{{\gamma }_{1}}({\boldsymbol{W}}_{1},{\mathcal{D}}_{1})}}{{Z}_{1}({\boldsymbol{W}}_{1})}\mathrm{log}\hfill \\ \hfill &amp; \quad \times \int \mathrm{d}{\boldsymbol{W}}_{2}\,{\text{e}}^{-{\beta }_{2}\left({\mathcal{L}}_{{\gamma }_{2}}({\boldsymbol{W}}_{2},{\mathcal{D}}_{2})+\frac{{\gamma }_{12}}{2}{\Vert}{\boldsymbol{W}}_{2}-{\boldsymbol{W}}_{1}{{\Vert}}_{2}^{2}\right)},\hfill \end{align*}

where the examples is ${\mathcal{D}}_{1},{\mathcal{D}}_{2}$ are characterised by a different variances in the irrelevant components.

This type of quantity is usually denoted as a ‘disordered’ partition function in statistical physics jargon, meaning that it is still dependent on a given realisation of the datasets, i.e. the source of disorder in this model. We want to characterise a typical realisation of this object, in the high-dimensional limit. However, because of its long-tailed statistics, the partition function turns out not to be a self-averaging quantity, i.e. its expectation over the dataset realisations will not correspond to the typical case scenario we are after. It is instead better to focus on the computation of the associated average free-entropy:

\begin{equation*}{\Phi}=\underset{N\to \infty }{\mathrm{lim}}\underset{{\beta }_{1},{\beta }_{2}\to \infty }{\mathrm{lim}}\frac{1}{{\beta }_{2}N}{\left\langle \mathrm{log}{\left\langle Z({\boldsymbol{W}}_{2},{\boldsymbol{W}}_{1};{\mathcal{D}}_{1},{\mathcal{D}}_{2})\right\rangle }_{{\boldsymbol{W}}_{1}}\right\rangle }_{{\mathcal{D}}_{1},{\mathcal{D}}_{2}}.\end{equation*}

What is immediately apparent is that we have to take the expectation of a logarithm, which is not tractable with rigorous mathematical methods. Moreover, we also have to average over the measure for ***W***_1_, which is also a complicated operation.

Fortunately, replica theory offers a method for approaching this calculation [40, 41]. The idea is to exploit two separate replica tricks:

• In order to evaluate the disorder average, the logarithm can be removed by replicating the second weight configuration, i.e. introducing *n* identical replicas ${\left\{{\boldsymbol{W}}_{2}^{a}\right\}}_{a=1}^{n}$, and extrapolating the final result from the *n* → 0 limit. This is based on the mathematical identity log *x* = lim_*n*→0_∂_*n*_ *x* ^*n*^.
• The average over the teacher can instead be computed by introducing $\tilde{n}-1$ non-interacting and a single interacting replica of the first weight configuration ${\left\{{\boldsymbol{w}}_{1}^{c}\right\}}_{c=1}^{\tilde{n}}$. Thus, only the *c* = 1 replica will enter the Gaussian prior in the student measure. The sought statistical average is again recovered in the limit $\tilde{n}\to 0$.

Because of the high-dimensional limit we are considering, all typical realisations of the teacher vector with a given sparsity *ρ* will yield an identical free-entropy. Thus, we can avoid averaging and instead fix a gauge ***W***_*T*,*i*_ = 1 for *i* = 1, …, *ρN* and ***W***_*T*,*i*_ = 0 elsewhere. In order to simplify the presentation, in the following we will assume that the datasets contain respectively *α*_1_ and *α*_2_ patterns, and that a curriculum ordering was employed, Δ_1_ < Δ_2_. Moreover, to avoid confusion with component and replica indices, we will denote with $\tilde{\boldsymbol{W}}={\boldsymbol{W}}_{1}$ and ***W*** = ***W***_2_, so that all quantities with a tilde refer to the optimisation on the first dataset.

After the described replication procedures, we get the following expression for the average free-entropy:

\begin{align*}\hfill {\Phi}&amp; =\frac{1}{N}\underset{n,\tilde{n}\to 0}{\mathrm{lim}}{\partial }_{n}\left\langle \underset{\tilde{\beta },\beta \to \infty }{\mathrm{lim}}\frac{1}{\beta }\int \prod\limits _{c=1}^{\tilde{n}}\mathrm{d}{\tilde{\boldsymbol{W}}}^{c}{e}^{-\frac{\tilde{\beta }{\gamma }_{1}}{2}{\Vert}{\tilde{\boldsymbol{W}}}^{c}{{\Vert}}_{2}^{2}}\prod\limits _{\mu =1}^{{\alpha }_{1}N}\right.\hfill \\ \hfill &amp; \quad \left.\times \prod\limits _{c=1}^{\tilde{n}}{e}^{-\frac{\beta }{2}\ell \left(\mathrm{sign}\left(\sum\limits _{i=1}^{\rho N}\frac{{x}_{i}^{\mu }}{\sqrt{N}}\right),\sigma \left(\sum\limits _{i=1}^{N}\frac{{\tilde{W}}_{i}^{c}{x}_{i}^{\mu }\left({{\Delta}}_{1}\right)}{\sqrt{N}}\right)\right)}\right.\hfill \\ \hfill &amp; \quad \times \int \prod\limits _{a=1}^{n}\mathrm{d}{\boldsymbol{W}}^{a}{e}^{-\frac{\beta {\gamma }_{2}}{2}{\Vert}{\boldsymbol{W}}^{a}{{\Vert}}_{2}^{2}}{e}^{-\frac{\beta {\gamma }_{12}}{2}{\Vert}{\boldsymbol{W}}^{a}-{\tilde{\boldsymbol{W}}}^{1}{{\Vert}}_{2}^{2}}\left.\prod\limits _{\mu =1}^{{\alpha }_{2}}\right.\hfill \\ \hfill &amp; \quad {\left.\times \prod\limits _{a}{e}^{-\frac{\beta }{2}\ell \left(\mathrm{sign}\left(\sum\limits _{i=1}^{\rho N}\frac{{x}_{i}^{\mu }}{\sqrt{N}}\right),\sigma \left(\sum\limits _{i=1}^{N}\frac{{W}_{i}^{a}{x}_{i}^{\mu }\left({{\Delta}}_{2}\right)}{\sqrt{N}}\right)\right)}\right\rangle }_{\left\{{\boldsymbol{x}}^{\mu }\right\}},\hfill \end{align*}

where $\ell (y,\hat{y})=\mathrm{log}(1+{e}^{-y\hat{y}})$ indicates the standard logistic loss. The next step is to explicitly compute the averages over the dataset realisations. Before doing that, we need to isolate the dependence of our expression on the patterns, and we achieve this by introducing Dirac’s *δ*-functions for the pre-activations. We will use the integral representation of the *δ*, with integration variables *u* for the teacher preactivations *λ* for the student preactivations:

\begin{align*}\hfill &amp; \frac{1}{N}\underset{n,\tilde{n}\to 0}{\mathrm{lim}}{\partial }_{n}\int \prod\limits _{c=1}^{\tilde{n}}\mathrm{d}{\tilde{\boldsymbol{W}}}^{c}{e}^{-\frac{\beta \lambda }{2}{\Vert}{\tilde{\boldsymbol{W}}}^{c}{{\Vert}}_{2}^{2}}\int \prod\limits _{a=1}^{\tilde{n}}\mathrm{d}{\boldsymbol{W}}^{a}{e}^{-\frac{\beta \lambda }{2}{\Vert}{\boldsymbol{W}}^{a}{{\Vert}}_{2}^{2}}{e}^{-\frac{\beta {\lambda }_{12}}{2}{\Vert}{\boldsymbol{W}}^{a}-{\tilde{\boldsymbol{W}}}^{1}{{\Vert}}_{2}^{2}}\hfill \\ \hfill &amp; \hspace{25.0pt}\quad \times \left\langle \int \prod\limits _{\mu }\frac{\mathrm{d}{\tilde{u}}_{1\mu }\mathrm{d}{\hat{\tilde{u}}}_{1\mu }}{2\pi }{\text{e}}^{\text{i}{\hat{\tilde{u}}}_{1\mu }\left({\tilde{u}}_{1\mu }-\sum\limits _{i=1}^{\rho N}\frac{{\left({\tilde{x}}_{1}\right)}_{i}^{\mu }}{\sqrt{N}}\right)}\int \prod\limits _{\mu ,c}\frac{\mathrm{d}{\tilde{\lambda }}_{1\mu }^{c}\mathrm{d}{\hat{\tilde{\lambda }}}_{1\mu }^{c}}{2\pi }\right.\hfill \\ \hfill &amp; \quad \hspace{25.0pt}\left.\times {\text{e}}^{\text{i}{\hat{\tilde{\lambda }}}_{1\mu }^{c}\left({\lambda }_{1\mu }^{c}-\sum\limits _{i=1}^{N}\frac{{\tilde{W}}_{i}^{c}{\left({\tilde{x}}_{1}\right)}_{i}^{\mu }}{\sqrt{N}}\right)}\int \prod\limits _{\mu }\frac{\mathrm{d}{u}_{2\mu }\mathrm{d}{\hat{u}}_{2\mu }}{2\pi }{\text{e}}^{\text{i}{\hat{u}}_{2\mu }\left({u}_{2\mu }-\sum\limits _{i=1}^{\rho N}\frac{{\left({x}_{2}\right)}_{i}^{\mu }}{\sqrt{N}}\right)}\right.\hfill \\ \hfill &amp; \quad \hspace{25.0pt}{\left.\times \int \prod\limits _{\mu ,a}\frac{d{\lambda }_{2\mu }^{a}\mathrm{d}{\hat{\lambda }}_{2\mu }^{a}}{2\pi }{\text{e}}^{\text{i}{\hat{\lambda }}_{2\mu }^{a}\left({\lambda }_{2\mu }^{a}-\sum\limits _{i=1}^{N}\frac{{W}_{i}^{a}{\left({x}_{2}\right)}_{i}^{\mu }}{\sqrt{N}}\right)}\right\rangle }_{\left\{{\boldsymbol{x}}^{\mu }\right\}}\ \hfill \\ \hfill &amp; \hspace{25.0pt}\quad \times \prod\limits _{\mu ,c}{e}^{-\frac{\beta }{2}\ell \left(\mathrm{sign}\left({\tilde{u}}_{1\mu }\right),\sigma \left({\tilde{\lambda }}_{1\mu }^{c}\right)\right)}\prod\limits _{\mu ,a}{e}^{-\frac{\beta }{2}\ell \left(\mathrm{sign}\left({u}_{2\mu }\right),\sigma \left({\lambda }_{2\mu }^{a}\right)\right)}.\hfill \end{align*}

Thus, the disorder average is now factorised and only involves exponential terms. Since the two datasets are independent now that we made the teacher explicit, we can take the averages over each one separately. In both cases we get:

\begin{align*}\hfill \left\langle .\right\rangle &amp; =\prod\limits _{i=1}^{\rho N}{\mathbb{E}}_{{\left({x}_{\text{rel}}\right)}_{i}^{\mu }}{\text{e}}^{-\text{i}\left(\frac{\hat{u}}{\sqrt{N}}+\sum\limits _{a}{\hat{\lambda }}_{a}^{\mu }\frac{{W}_{i}^{a}}{\sqrt{N}}\right){\left({x}_{\text{rel}}\right)}_{i}^{\mu }}\prod\limits _{i=\rho N+1}^{N}{\mathbb{E}}_{{\left({x}_{\text{irr}}\right)}_{i}^{\mu }}{\text{e}}^{-\text{i}\left(\sum\limits _{a}{\hat{\lambda }}_{a}^{\mu }\frac{{W}_{i}^{a}}{\sqrt{N}}\right){\left({x}_{\text{irr}}\right)}_{i}^{\mu }}\hfill \\ \hfill &amp; =\prod\limits _{i=1}^{\rho N}\left(1-i\left(\frac{\hat{u}}{\sqrt{N}}+\sum\limits _{a}{\hat{\lambda }}_{a}^{\mu }\frac{{W}_{i}^{a}}{\sqrt{N}}\right)\bar{{x}_{\text{rel}}}-\frac{1}{2}{\left(\frac{\hat{u}}{\sqrt{N}}+\sum\limits _{a}{\hat{\lambda }}_{a}^{\mu }\frac{{W}_{i}^{a}}{\sqrt{N}}\right)}^{2}\mathrm{Var}\left({x}_{\text{rel}}\right)\right)\hfill \\ \hfill &amp; \quad \times \prod\limits _{i=\rho N+1}^{N}\left(1-i\sum\limits _{a}{\hat{\lambda }}_{a}^{\mu }\frac{{W}_{i}^{a}}{\sqrt{N}}\bar{{x}_{\text{irr}}}-\frac{1}{2}{\left(\sum\limits _{a}{\hat{\lambda }}_{a}^{\mu }\frac{{W}_{i}^{a}}{\sqrt{N}}\right)}^{2}\mathrm{Var}({x}_{\text{irr}})\right)\hfill \end{align*}

\begin{align*}\hfill &amp; =\prod\limits _{i=1}^{\rho N}\left(1-\frac{1}{2N}{\left({\hat{u}}^{\mu }\right)}^{2}-\frac{1}{N}\sum\limits _{a}{\hat{u}}^{\mu }{\hat{\lambda }}_{a}^{\mu }{W}_{i}^{a}-\frac{1}{2N}\sum\limits _{ab}{\hat{\lambda }}_{a}^{\mu }{\hat{\lambda }}_{b}^{\mu }{W}_{i}^{a}{W}_{i}^{b}\right)\hfill \\ \hfill &amp; \quad \times \prod\limits _{i=\rho N+1}^{N}\left(1-\frac{{{\Delta}}^{\mu }}{2N}\sum\limits _{ab}{\hat{\lambda }}_{a}^{\mu }{\hat{\lambda }}_{b}^{\mu }{W}_{i}^{a}{W}_{i}^{b}\right)\hfill \\ \hfill &amp; ={e}^{-\frac{1}{2}\sum\limits _{ab}{\hat{\lambda }}_{a}^{\mu }{\hat{\lambda }}_{b}^{\mu }\left(\frac{{\sum }_{i=1}^{\rho N}{W}_{i}^{a}{W}_{i}^{b}}{N}+{\Delta}\frac{{\sum }_{i=\rho N+1}^{N}{W}_{i}^{a}{W}_{i}^{b}}{N}\right)-\frac{\rho }{2}{\left({\hat{u}}^{\mu }\right)}^{2}-{\hat{u}}^{\mu }\sum\limits _{a}{\hat{\lambda }}_{a}^{\mu }\frac{{\sum }_{i=1}^{\eta N}{W}_{i}^{a}}{N}}.\hfill \end{align*}

This expression suggests what are the order parameters that capture the interactions of the model, namely:

• The teacher–student overlap at the end of the first learning phase: ${\tilde{R}}^{c}=\frac{{\sum }_{i=1}^{\rho N}{\tilde{W}}_{i}^{c}}{N}$.
• The teacher–student overlap at the end of the second learning phase: ${R}^{a}=\frac{{\sum }_{i=1}^{\rho N}{W}_{i}^{a}}{N}$.
• The norm of the student after the first stage, decomposed into relevant/irrelevant parts: ${\tilde{Q}}_{r}^{cd}=\frac{{\sum }_{i=1}^{\rho N}{\tilde{W}}_{i}^{c}{\tilde{W}}_{i}^{d}}{N}$, ${\tilde{Q}}_{i}^{cd}=\frac{{\sum }_{i=\rho N+1}^{N}{\tilde{W}}_{i}^{c}{\tilde{W}}_{i}^{d}}{N}$.
• The norm of the student after the second stage, decomposed into relevant/irrelevant parts: ${Q}_{r}^{ab}=\frac{{\sum }_{i=1}^{\rho N}{W}_{i}^{a}{W}_{i}^{b}}{N}$, ${Q}_{i}^{ab}=\frac{{\sum }_{i=\rho N+1}^{N}{W}_{i}^{a}{W}_{i}^{b}}{N}$.

Therefore, after introducing these definitions by means of Dirac’s *δ*-functions, we can rewrite our replicated expression as:

\begin{align*}\hfill {{\Omega}}^{n}&amp; =\int \prod\limits _{c}\frac{\mathrm{d}{\tilde{R}}^{c}\mathrm{d}{\hat{\tilde{R}}}^{c}}{2\pi /N}\int \prod\limits _{a}\frac{\mathrm{d}{R}^{a}\mathrm{d}{\hat{R}}^{a}}{2\pi /N}\int \prod\limits _{cd}\frac{\mathrm{d}{\tilde{Q}}_{r}^{cd}\mathrm{d}{\hat{\tilde{Q}}}_{r}^{cd}}{2\pi /N}\int \prod\limits _{cd}\frac{\mathrm{d}{\tilde{Q}}_{i}^{cd}\mathrm{d}{\hat{\tilde{Q}}}_{i}^{cd}}{2\pi /N}\hfill \\ \hfill &amp; \quad \times \int \prod\limits _{ab}\frac{\mathrm{d}{Q}_{r}^{ab}\mathrm{d}{\hat{Q}}_{r}^{ab}}{2\pi /N}\int \prod\limits _{ab}\frac{\mathrm{d}{Q}_{i}^{ab}\mathrm{d}{\hat{Q}}_{i}^{ab}}{2\pi /N}{G}_{i}\,{G}_{S}{\left(\hat{\tilde{R}},\hat{R},{\hat{\tilde{Q}}}_{r},{\hat{Q}}_{r}\right)}^{\rho N}\hfill \\ \hfill &amp; \quad \times {G}_{S}{\left(0,0,{\tilde{Q}}_{i},{Q}_{i}\right)}^{\left(1-\rho \right)N}{G}_{E}{\left({{\Delta}}_{1},{\tilde{Q}}_{r},{\tilde{Q}}_{i},\tilde{R},\tilde{n}\right)}^{{\alpha }_{1}N}{G}_{E}{\left({{\Delta}}_{2},{Q}_{r},{Q}_{i},R,n\right)}^{{\alpha }_{2}N}\hfill \end{align*}

where we introduced interaction, entropic and energetic potentials:

\begin{align*}\hfill {G}_{i}&amp; =\mathrm{exp}\left(-N\left(\sum\limits _{c}{\hat{\tilde{m}}}^{c}{\tilde{m}}^{c}+\sum\limits _{a}{\hat{m}}^{a}{m}^{a}+\sum\limits _{cd}{\hat{\tilde{Q}}}_{r}^{cd}{\tilde{Q}}_{r}^{cd}+\sum\limits _{cd}{\hat{\tilde{Q}}}_{i}^{cd}{\tilde{Q}}_{i}^{cd}\right.\right.\hfill \\ \hfill &amp; \quad \left.\left.+\sum\limits _{ab}{\hat{Q}}_{r}^{ab}{Q}_{r}^{ab}+\sum\limits _{ab}{\hat{Q}}_{i}^{ab}{Q}_{i}^{ab}\right)\right)\hfill \end{align*}

\begin{align*}\hfill {G}_{S}\left(\tilde{R},R,\tilde{Q},Q\right)&amp; =\int \prod\limits _{c}\left[\mathrm{d}{\tilde{W}}^{c}{e}^{-\frac{\beta \gamma }{2}{\left({\tilde{W}}^{c}\right)}^{2}}\right]{e}^{-\frac{n\beta {\gamma }_{12}}{2}{({\tilde{W}}^{1})}^{2}}\,\int \prod\limits _{a}\left[\mathrm{d}{W}^{a}{e}^{-\frac{\beta \left(\gamma +{\gamma }_{12}\right)}{2}{\left({W}^{a}\right)}^{2}}\right]\hfill \\ \hfill &amp; \quad \times \mathrm{exp}\left(\sum\limits _{c}{\hat{\tilde{R}}}^{c}{\tilde{W}}^{c}+\sum\limits _{a}{\hat{R}}^{a}{W}^{a}+\sum\limits _{cd}{\hat{\tilde{Q}}}^{cd}{\tilde{W}}^{c}{\tilde{W}}^{d}\right.\hfill \\ \hfill &amp; \quad \left.+\sum\limits _{ab}{\hat{Q}}^{ab}{W}^{a}{W}^{b}+\beta {\gamma }_{12}{W}^{a}{\tilde{W}}^{1}\right)\hfill \end{align*}

\begin{align*}\hfill {G}_{E}\left({\Delta},{Q}_{r},{Q}_{i},m,n\right)&amp; =\int \frac{\mathrm{d}u\mathrm{d}\hat{u}}{2\pi }{e}^{iu\hat{u}}{e}^{-\frac{\rho }{2}{\left(\hat{u}\right)}^{2}}\int \prod\limits _{a=1}^{n}\frac{d{\lambda }^{a}\mathrm{d}{\hat{\lambda }}^{a}}{2\pi }{\text{e}}^{\text{i}{\lambda }^{a}{\hat{\lambda }}^{a}}\hfill \\ \hfill &amp; \quad \times {e}^{-\frac{1}{2}\sum\limits _{ab}{\hat{\lambda }}_{a}{\hat{\lambda }}_{b}\left({Q}_{r}^{ab}+{\Delta}{Q}_{i}^{ab}\right)-\hat{u}\sum\limits _{a}{\hat{\lambda }}_{a}{R}^{a}-\frac{\beta }{2}\ell \left(u,{\lambda }^{a}\right)}.\hfill \end{align*}

*Replica symmetric ansatz*. The replica trick allowed us to express the average free-entropy as a function of the overlap order parameters. However, these objects are *n* × *n* matrices or *n*-dimensional vectors and in principle we have to average over all their possible realisations. Fortunately, the integrand function is exponential in *N* and in the thermodynamic limit *N* → ∞ the integrals are dominated by the extremisers of the action, and thus can be approximated with the saddle-point method. Still, we need a guess for how to parameterise these order parameters. The simplest possible ansatz, which turns out to be the correct one in convex problems as the one at hand, is the so-called replica symmetric (RS) ansatz, given by:

• ${\tilde{R}}^{c}=\tilde{R}$.
• *R* ^*a*^ = *R*.
• ${\tilde{Q}}_{r/i}^{cd}={\tilde{q}}_{r/i}$, for *c* ≠ *d*; ${\tilde{Q}}_{r/i}^{cd}={\tilde{Q}}_{r/i}$ for *c* = *d*.
• ${Q}_{r/n}^{ab}={q}_{r/n}$ for *a* ≠ *b*; ${Q}_{r/n}^{ab}={Q}_{r/n}$ for *a* = *b*.

We also perform a Wick rotation $-i{\hat{Q}}_{ac,bd}\to {\hat{Q}}_{ac,bd}$ in order to deal with real valued conjugate parameters and pose a similar ansatz for them. In the next paragraph we will compute the three terms separately, and finally put them together in the expression for the RS free-entropy.

*Interaction term*. We start by evaluating the interaction term, or better its normalised logarithm ${g}_{i}={\mathrm{lim}}_{\tilde{n}\to 0}\mathrm{log}{G}_{i}/(nN)$:

\begin{align*}\hfill {g}_{i}&amp; =-\underset{\tilde{n}\to 0}{\mathrm{lim}}\frac{1}{n}\left(\tilde{n}\hat{\tilde{R}}\tilde{R}+n\hat{R}R+\tilde{n}\left(\frac{{\hat{\tilde{Q}}}_{r}{\tilde{Q}}_{r}}{2}+\frac{{\hat{\tilde{Q}}}_{i}{\tilde{Q}}_{i}}{2}\right)+\frac{\tilde{n}(\tilde{n}-1)}{2}\left({\hat{\tilde{q}}}_{r}{\tilde{q}}_{r}+{\hat{\tilde{q}}}_{i}{\tilde{q}}_{i}\right)\right.\hfill \\ \hfill &amp; \quad \left.+n\left(\frac{{\hat{Q}}_{r}{Q}_{r}}{2}+\frac{{\hat{Q}}_{i}{Q}_{i}}{2}\right)+\frac{n(n-1)}{2}\left({\hat{q}}_{r}{q}_{r}+{\hat{q}}_{i}{q}_{i}\right)\right)\hfill \end{align*}

\begin{equation*}=-\left(\hat{R}R+\frac{\left({\hat{Q}}_{r}{Q}_{r}+{\hat{Q}}_{i}{Q}_{i}\right)}{2}-\frac{1}{2}\left({\hat{q}}_{r}{q}_{r}+{\hat{q}}_{i}{q}_{i}\right)\right)\end{equation*}

In order to recover the optimisation problems entailed in the curriculum procedure, we now have to consider the zero temperature limit of this expression. When *β* → ∞, the order parameters follow non-trivial scaling laws:

• $\hat{Q}\to {\beta }^{2}\hat{Q}+\mathcal{O}\left(\beta \right)$, $\hat{q}\to {\beta }^{2}\hat{Q}$
• $(\hat{Q}-\hat{q})\to -\beta \delta \hat{Q}$
• $\hat{R}\to \beta \hat{R}$
• *Q* − *q* = *δQ*/*β*

and similarly for the tilde parameters. Intuitively, looking at the last scaling law, we see that as the measure gets focused on the single minimiser of the loss, the overlap between different replicas *q* rapidly converges to the norm *Q*. Moreover, the scaling with the inverse temperature of the conjugate parameters prevents the interaction term from becoming sub-dominant in the saddle-point. If we substitute the rescaled parameters in the above expression we obtain:

\begin{equation*}{g}_{i}=-\beta \left(\hat{R}R+\frac{1}{2}\left({\hat{Q}}_{r}\delta {Q}_{r}-\delta {\hat{Q}}_{r}{Q}_{r}\right)+\frac{1}{2}\left({\hat{Q}}_{i}\delta {Q}_{i}-\delta {\hat{Q}}_{i}{Q}_{i}\right)\right).\end{equation*}

*Entropic term*. We can now compute a similar quantity for the entropic potential, ${g}_{i}=\frac{{\mathrm{lim}}_{n\to 0}}{n}\mathrm{log}\,{G}_{S}\left(\tilde{R},R,\tilde{Q},Q\right)$. The general expression we will obtain can be specialised to the two cases $\left(\left\{\tilde{R},R,{\tilde{Q}}_{r},{Q}_{r}\right\},\left\{0,0,{\tilde{Q}}_{i},{Q}_{i}\right\}\right)$ appearing in the free-entropy. After substituting the RS ansatz we find:

\begin{align*}\hfill {g}_{S}&amp; =\underset{\tilde{n}\to 0}{\mathrm{lim}}\frac{1}{n}\mathrm{log}\int \prod\limits _{c}\left[\mathrm{d}{\tilde{W}}^{c}{e}^{-\frac{\beta \gamma }{2}{\left({\tilde{W}}^{c}\right)}^{2}}\right]{e}^{-\frac{n\beta {\gamma }_{12}}{2}{({\tilde{W}}^{1})}^{2}}\int \prod\limits _{a}\left[\mathrm{d}{W}^{a}{e}^{-\frac{\beta \left(\gamma +{\gamma }_{12}\right)}{2}{\left({W}^{a}\right)}^{2}}\right]\hfill \\ \hfill &amp; \quad \times \mathrm{exp}\left(\hat{\tilde{R}}\sum\limits _{c}{\tilde{W}}^{c}+\hat{R}\sum\limits _{a}{W}^{a}+\frac{1}{2}\left(\hat{\tilde{Q}}-\hat{\tilde{q}}\right)\sum\limits _{c}{\left({\tilde{W}}^{c}\right)}^{2}+\frac{\hat{\tilde{q}}}{2}{\left(\sum\limits _{c}{\tilde{W}}^{c}\right)}^{2}\right.\hfill \\ \hfill &amp; \quad \left.+\frac{1}{2}\left(\hat{Q}-\hat{q}\right)\sum\limits _{a}{\left({W}^{a}\right)}^{2}+\frac{\hat{q}}{2}{\left(\sum\limits _{a}{W}^{a}\right)}^{2}+\beta {\gamma }_{12}\sum\limits _{a}{\tilde{W}}^{1}{W}^{a}\right)\hfill \\ \hfill &amp; =\underset{\tilde{n}\to 0}{\mathrm{lim}}\frac{1}{n}\mathrm{log}\int \mathcal{D}z\int \mathcal{D}\tilde{z}\int \prod\limits _{c}\left[\mathrm{d}{\tilde{W}}^{c}{e}^{-\frac{\beta \gamma }{2}{\left({\tilde{W}}^{c}\right)}^{2}}\right]{e}^{-\frac{n\beta {\gamma }_{12}}{2}{({\tilde{W}}^{1})}^{2}}\int \prod\limits _{a}\mathrm{d}{W}^{a}\hfill \\ \hfill &amp; \quad \times {e}^{-\frac{\beta \left(\gamma +{\gamma }_{12}\right)}{2}{\left({W}^{a}\right)}^{2}}\mathrm{exp}\left(\frac{1}{2}\left(\hat{\tilde{Q}}-\hat{\tilde{q}}\right)\sum\limits _{c}{\left({\tilde{W}}^{c}\right)}^{2}+\frac{1}{2}\left(\hat{Q}-\hat{q}\right)\sum\limits _{a}{\left({W}^{a}\right)}^{2}\right.\hfill \\ \hfill &amp; \quad +\left.\left(\hat{\tilde{R}}+\sqrt{\hat{\tilde{q}}}\tilde{z}\right)\sum\limits _{c}{\tilde{W}}^{c}+\left(\hat{R}+\beta {\gamma }_{12}{W}_{1}+\sqrt{\hat{q}}z\right)\sum\limits _{a}{W}^{a}\right)\hfill \end{align*}

\begin{equation*}=\int \mathcal{D}z\int \mathcal{D}\tilde{z}\frac{\int \mathrm{d}\tilde{W}{e}^{-\frac{1}{2}(\beta \tilde{\gamma }-(\hat{\tilde{Q}}-\hat{\tilde{q}})){\tilde{W}}^{2}+(\hat{\tilde{R}}+\sqrt{\hat{\tilde{q}}}\tilde{z})\tilde{W}}\,\mathrm{log}\left(\int \mathrm{d}W\,{e}^{-\frac{1}{2}\left(\beta \left(\gamma +{\gamma }_{12}\right)-(\hat{Q}-\hat{q})\right){W}^{2}+\left(\hat{R}+\beta {\gamma }_{12}{W}_{1}+\sqrt{\hat{q}}z\right)W}\right)}{\int \mathrm{d}\tilde{W}{e}^{-\frac{1}{2}(\beta \tilde{\gamma }-(\hat{\tilde{Q}}-\hat{\tilde{q}})){\tilde{W}}^{2}+(\hat{\tilde{R}}+\sqrt{\hat{\tilde{q}}}\tilde{z})\tilde{W}}}.\end{equation*}

In the zero-temperature limit, we consider the same rescaling of the order parameters we described above. The integrals over the weights become an extremum operation:

\begin{equation*}{g}_{s}=\underset{\beta \to \infty }{\mathrm{lim}}\beta \int \mathcal{D}z\int \mathcal{D}\tilde{z}{M}_{s}^{\star },\end{equation*}

where:

\begin{equation*}{M}_{s}^{\star }={\mathrm{max}}_{W}\left\{-\frac{1}{2}\left(\left(\gamma +{\gamma }_{12}\right)+\delta \hat{Q}\right){W}^{2}+\left(\hat{R}+{\gamma }_{12}{\tilde{W}}^{\star }+\sqrt{\hat{Q}}z\right)W\right\}\end{equation*}

\begin{equation*}\quad =\frac{1}{2}\frac{{\left(\hat{R}+{\gamma }_{12}{\tilde{W}}^{\star }+\sqrt{\hat{Q}}z\right)}^{2}}{\left(\gamma +{\gamma }_{12}\right)+\delta \hat{Q}}\end{equation*}

and where: ${\tilde{W}}^{\star }={\mathrm{a}\mathrm{r}\mathrm{g}\mathrm{m}\mathrm{a}\mathrm{x}}_{\tilde{W}}\left\{-\frac{1}{2}(\tilde{\gamma }+\,\delta \hat{\tilde{Q}}){\tilde{W}}^{2}+(\hat{\tilde{R}}+\sqrt{\hat{\tilde{Q}}}\tilde{z})\tilde{W}\right\}=\frac{\hat{\tilde{R}}+\sqrt{\hat{\tilde{Q}}}\tilde{z}}{\tilde{\gamma }+\delta \hat{\tilde{Q}}}$.

Finally also the $\int \mathcal{D}z\int \mathcal{D}\tilde{z}$ integrations can be carried out, giving:

\begin{equation*}\beta \int \mathcal{D}z\int \mathcal{D}\tilde{z}{M}_{s}^{\star }=\beta \int \mathcal{D}z\int \mathcal{D}\tilde{z}\frac{1}{2}\frac{{\left(\hat{R}+{\gamma }_{12}\frac{\hat{\tilde{R}}+\sqrt{\hat{\tilde{Q}}}\tilde{z}}{\tilde{\gamma }+\,\delta \hat{\tilde{Q}}}+\sqrt{\hat{Q}}z\right)}^{2}}{\left(\gamma +{\gamma }_{12}\right)+\delta \hat{Q}}\end{equation*}

\begin{equation*}=\frac{\beta }{2}\frac{{\left(\hat{R}+\hat{\tilde{R}}\frac{{\gamma }_{12}}{\tilde{\gamma }+\delta \hat{\tilde{Q}}}\right)}^{2}+{\left(\frac{{\gamma }_{12}\sqrt{\hat{\tilde{Q}}}}{\tilde{\gamma }+\delta \hat{\tilde{Q}}}\right)}^{2}+\hat{Q}}{\left(\gamma +{\gamma }_{12}\right)+\delta \hat{Q}}.\end{equation*}

So, specialising to the two terms that appear in the free-entropy we get:

\begin{align*}\hfill {g}_{S}({\gamma }_{1},{\gamma }_{2},{\gamma }_{12})&amp; =\rho \,{g}_{s}\left(\tilde{R},R,{\tilde{Q}}_{r},{Q}_{r}\right)+\left(1-\rho \right)\,{g}_{s}\left(0,0,{\tilde{Q}}_{i},{Q}_{i}\right)\hfill \\ \hfill &amp; =\frac{\beta }{2}\left(\rho \frac{{\left(\hat{R}+\hat{\tilde{R}}\frac{{\gamma }_{12}}{{\gamma }_{1}+\delta {\hat{\tilde{Q}}}_{r}}\right)}^{2}+{\left(\frac{{\gamma }_{12}{\sqrt{\hat{\tilde{Q}}}}_{r}}{{\gamma }_{1}+\delta {\hat{\tilde{Q}}}_{r}}\right)}^{2}+{\hat{Q}}_{r}}{\left({\gamma }_{2}+{\gamma }_{12}\right)+\delta {\hat{Q}}_{r}}\right.\,\left.+\left(1-\rho \right)\frac{{\left(\frac{{\gamma }_{12}\sqrt{{\hat{\tilde{Q}}}_{i}}}{{\gamma }_{1}+\delta {\hat{\tilde{Q}}}_{i}}\right)}^{2}+{\hat{Q}}_{i}}{\left({\gamma }_{2}+{\gamma }_{12}\right)+\delta {\hat{Q}}_{i}}\right).\hfill \end{align*}

*Energetic term*. Since one of the two energetic terms appearing in the replicated free-energy depends on the $\tilde{n}$ replicas of the first weight configuration, and there is no interaction, we can take the $\tilde{n}\to 0$ limit directly. Therefore we only have to evaluate the other contribution (dependent on the *n* replicas of the second weight configuration). Defining *Q* = *Q*_*r*_ + Δ*Q*_*i*_, *Q* = *Q*_*r*_ + Δ*Q*_*i*_, we evaluate ${g}_{E}={\mathrm{lim}}_{n\to 0}\frac{1}{n}\mathrm{log}({G}_{E})$ in the RS ansatz:

\begin{align*}\hfill {g}_{E}&amp; =\underset{n\to 0}{\mathrm{lim}}\frac{1}{n}\mathrm{log}\int \frac{\mathrm{d}u\mathrm{d}\hat{u}}{2\pi }{e}^{iu\hat{u}}{e}^{-\frac{\rho }{2}{\left(\hat{u}\right)}^{2}}\int \prod\limits _{a}\left(\frac{d{\lambda }^{a}\mathrm{d}{\hat{\lambda }}^{a}}{2\pi }{\text{e}}^{\text{i}{\lambda }^{a}{\hat{\lambda }}^{a}}\right)\hfill \\ \hfill &amp; \quad \times {e}^{-\frac{1}{2}\left(Q-q\right)\sum\limits _{a}{\left({\hat{\lambda }}_{a}\right)}^{2}-\frac{1}{2}q{\left(\sum\limits _{a}{\hat{\lambda }}_{a}\right)}^{2}-\hat{u}R\sum\limits _{a}{\hat{\lambda }}_{a}-\beta \sum\limits _{a}\ell \left(u,{\lambda }^{a}\right)}\hfill \end{align*}

\begin{align*}\hfill &amp; =\underset{n\to 0}{\mathrm{lim}}\frac{1}{n}\mathrm{log}\int \frac{\mathrm{d}u}{\sqrt{2\pi \rho }}\int \prod\limits _{a}\left(\frac{d{\lambda }^{a}\mathrm{d}{\hat{\lambda }}^{a}}{2\pi }{\text{e}}^{\text{i}{\lambda }^{a}{\hat{\lambda }}^{a}}\right)\hfill \\ \hfill &amp; \quad \times {e}^{-\frac{1}{2}\left(Q-q\right)\sum\limits _{a}{\left({\hat{\lambda }}_{a}\right)}^{2}-\frac{1}{2}q{\left(\sum\limits _{a}{\hat{\lambda }}_{a}\right)}^{2}-\beta \sum\limits _{a}\ell \left(u,{\lambda }^{a}\right)-\frac{1}{2\rho }{\left(u+i\,R\sum\limits _{a}{\hat{\lambda }}_{a}\right)}^{2}}\hfill \end{align*}

\begin{align*}\hfill &amp; =\underset{n\to 0}{\mathrm{lim}}\frac{1}{n}\mathrm{log}\int \mathcal{D}{z}_{0}\int \mathcal{D}u{\left\{\int \mathcal{D}\lambda {\text{e}}^{-\beta \,\ell \left(\sqrt{\rho }\,u,\sigma \left(\sqrt{\left(Q-q\right)}\lambda +\sqrt{q-\frac{{m}^{2}}{\rho }}{z}_{0}+\frac{m}{\sqrt{\rho }}u\right)\right)}\right\}}^{n}\hfill \\ \hfill &amp; =\int \mathcal{D}{z}_{0}\int \mathcal{D}u\mathrm{log}\int \mathcal{D}\lambda {\text{e}}^{-\beta \,\ell \left(\sqrt{\rho }\,u,\sigma \left(\sqrt{Q-q}\lambda +\sqrt{q-\frac{{m}^{2}}{\rho }}{z}_{0}+\frac{m}{\sqrt{\rho }}u\right)\right)}.\hfill \end{align*}

So in the *β* → ∞ limit, with the proper rescalings, we get:

\begin{equation*}{g}_{E}=\beta \int \mathcal{D}z\int \mathcal{D}u\,{M}_{E}^{\star },\end{equation*}

where:

\begin{align*}\hfill {M}_{E}^{\star }&amp; ={\mathrm{max}}_{\lambda }-\frac{{\lambda }^{2}}{2}-\ell \left(\mathrm{sign}\left(\sqrt{\rho }\,u\right),\sigma \left(\sqrt{\delta {Q}_{r}+{\Delta}\delta {Q}_{i}}\lambda \right.\right.\,\left.\left.\ +\sqrt{{Q}_{r}+{\Delta}{Q}_{i}-\frac{{R}^{2}}{\rho }}z+\frac{R}{\sqrt{\rho }}u\right)\right).\hfill \end{align*}

*RS free-entropy*. Finally, assuming the we can write down the RS free-entropy for the curriculum ordering as:

\begin{align*}\hfill {\Phi}/\beta &amp; =-\mathrm{e}\mathrm{x}\mathrm{t}\mathrm{r}\left(\hat{R}R+\frac{1}{2}\left({\left(\hat{Q}\delta Q-\delta \hat{Q}Q\right)}_{r}+{\left(\hat{Q}\delta Q-\delta \hat{Q}Q\right)}_{i}\right)\right)\hfill \\ \hfill &amp; \quad +\,{g}_{S}({\gamma }_{1},{\gamma }_{2},{\gamma }_{12})+{\alpha }_{2}\,{g}_{E}\left({{\Delta}}_{2}\right),\hfill \end{align*}

where *g*_*S*_ is defined in equation (B.21) and *g*_*E*_ is defined in equation (B.25). The order parameters for the teacher system are obtained independently from identical equations, after substituting *λ*_1_ → 0, *λ*_2_ =→ *λ*_1_ and *λ*_12_ → 0, *α*_2_ → *α*_1_ and Δ_2_ → Δ_1_, and after adding a tilde to the remaining parameters.

The saddle-point equations, yielding at convergence the asymptotic prediction for the order parameters, can be found by posing stationarity conditions for the free-entropy with respect to all overlaps.

Note that, if instead of the simple setting just considered, where the data slice in the second stage has homogeneous variance for the irrelevant components, there are multiple subsets with different sizes and variances, the only variation in the free-entropy is in the energetic contribution. In general one will have a sum:

\begin{equation*}\sum\limits _{s}\,{\alpha }_{s}{g}_{E}({{\Delta}}_{s})\end{equation*}

over each of these subsets.

Moreover, if instead of two stages we consider multiple learning stages, the free-entropy for each successive step has an identical form, and one only has to substitute the tilde parameters with the order parameters obtained at the previous step. Note that the simplicity of nesting stages in this problem is connected to the convexity of this learning setting. Generally, adding more steps would increase the complexity of the calculation considerably.

*Generalisation error*. With the saddle-point values for the order parameters, one can easily evaluate the generalisation error on new datapoints, which is the measure of performance we are employing in the main. This performance can be obtained as:

\begin{equation*}1-{{\epsilon}}_{\mathrm{g}}={\left\langle {\Theta}\left(\left(\frac{{\boldsymbol{W}}_{T}\cdot x}{\sqrt{N}}\right)\left(\frac{{\boldsymbol{W}}_{2}\cdot x}{\sqrt{N}}\right)\right)\right\rangle }_{x({\Delta})}\end{equation*}

where Δ is the variance of the irrelevant components for the new pattern. A shortcut for evaluating this expression is to insert the order parameters in the expression through Dirac’s *δ*s. After a straightforward calculation, along the same lines of the one presented above, one obtains:

\begin{equation*}{{\epsilon}}_{\mathrm{g}}=\frac{1}{\pi }\mathrm{arccos}\left(\frac{R}{\sqrt{\rho \left({Q}_{r}+{\Delta}{Q}_{i}\right)}}\right).\end{equation*}

Of course, the generalisation accuracy is just the complementary quantity 1 − *ϵ*_g_.

## Appendix C. Additional results on sparsity

We complement the discussion on the importance of sparsity, section 4, with the comparison with other learning protocols. Observe that anti-curriculum suffers the same issue of the curriculum method for sufficiently large fractions of relevant features *ρ*. In that regime, the splitting becomes sub-optimal because the solution found in the splitting does not provide enough information to help the other phase of learning. Consequently, the network is forced to set neglect the information in the batch in favour of exploring solutions further away from that one. This is outperform by standard learning, where all the bits of information are used (figure C.1).

## Appendix D. Simulations on CIFAR10

**Task design**. Because a sparse set of relevant features is crucial to observing curriculum effects in our model, we created a task based on real data that has this property. In particular we create 32 × 64 pixel input examples by concatenating two images side-by-side from the CIFAR10 dataset (figure ??). The correct output label is given by the label of the image on the left, while the image on the right is an irrelevant distractor. To vary difficulty, we scale the contrast of the irrelevant image. This dataset is meant to instantiate a simple example of learning an object classification amidst clutter. We emphasise that, as in our synthetic data model, each training sample always contains the same relevant and distractor images (i.e., we are not considering a data augmentation setting where each relevant image appears with many non-relevant images). To ensure no cross-contamination of training and testing samples, the distractor images for the training and test sets are drawn only from the same set.

**Model architecture and training regime**. We train a single layer network with cross entropy loss (i.e. softmax regression), implemented in Pytorch Lightning by modifying the MIT-licensed PyTorch_CIFAR10 repository (https://zenodo.org/record/4431043#.YLmz6zZKhsA) to ensure that training parameters accord with standard practice. Networks were trained with SGD and Nesterov momentum, under default parameters: a learning rate of 1*e* − 2, momentum parameter 0.9, batch size 256, and 100 epochs. The learning rate was annealed according to the ‘WarmUpCosine’ schedule used in PyTorch_CIFAR10, which linearly reduces the learning rate over the first 30% of training steps before switching to a cosine shaped schedule on the remainder.

**Experiment details and hyperparameter optimisation**. For the first phase of training, we used dataset sizes in 10 equal steps between 1000 and 50 000. For the second phase, we used nine dataset sizes in 9 equal steps between 5333 and 48 000. We optimised hyperparameters in each phase separately. In the first phase, we evaluated all combinations of initialisation scales of {0, 0.2, 0.5, 1.0}, weight decay parameters of {0, 0.2, 0.5, 1.0, 2.0}, and curriculum policy, for five random seeds. In the second phase, for each random seed and curriculum condition, we continued training from the best-performing model obtained in the first phase. We trained all combinations of five elastic penalties log spaced between 1*e* − 3 and 1*e*2, and weight decay parameters {0, 0.2, 0.5}. We then compute the best performing model for each seed and take the mean over seeds. Finally, to evaluate the no-curriculum performance, we train shuffled dataset models with initialisation scales {0, 0.2, 0.5, 1.0} and weight decay parameters {0, 0.2, 0.5}. For visualisation purposes, we used nearest-neighbors interpolation in the phase portrait to provide values for all points used in the synthetic experiments. Experiments were run on V100 GPUs and required approximately 10 000 GPU hours (including debugging and development), or $\approx 1110$ kg CO_2_ equation according to the MachineLearning Impact calculator of Lacoste *et al*, 2019.

## Appendix E. Speed-up theory vs simulations

As remarked in the main text, one of the advantages of the theoretical analysis is a huge speed-up in the time to collect the results, without need of averaging to reduce the fluctuations. In this section, we briefly report a comparison between the time required for the lines from theory and simulations shown in the main text.

In order to obtain figure 1(c), a single run of the ODE equations takes 2 milliseconds and a run of the simulations takes 500 milliseconds. The figure is however obtained optimizing over all the hyperparameters (learning rate, initialization, weight decay) totalling 400 milliseconds for the analytical solution; while, due to noise, simulation results for a single set of hyperparameters requires averaging 5000 realizations totalling 41 min. We note that we did the hyperparameter optimization only once using the theoretical framework and then used the optima in the simulations in order to save compute time. The best comparison should therefore be done for a fixed set of hyperparameters and gives 2 milliseconds vs 41 min. Overall, the analytical solution is between 2 and 6 orders of magnitude faster.
